# Matriglycan maintains t-tubule structural integrity in cardiac muscle

**DOI:** 10.1073/pnas.2402890121

**Published:** 2024-05-21

**Authors:** Jeffrey M. Hord, Mary E. Anderson, Sally J. Prouty, Shelly Melton, Zeita Gastel, Kathy Zimmerman, Robert M. Weiss, Kevin P. Campbell

**Affiliations:** ^a^HHMI, University of Iowa, Iowa City, IA 52242; ^b^Senator Paul D. Wellstone Muscular Dystrophy Specialized Research Center, University of Iowa, Iowa City, IA 52242; ^c^Department of Molecular Physiology and Biophysics, Roy J. and Lucille A. Carver College of Medicine, University of Iowa, Iowa City, IA 52242; ^d^Department of Neurology, Roy J. and Lucille A. Carver College of Medicine, University of Iowa, Iowa City, IA 52242; ^e^Division of Cardiology, Department of Internal Medicine, Carver College of Medicine, University of Iowa, Iowa City, IA 52242; ^f^Abboud Cardiovascular Research Center, Carver College of Medicine, Department of Internal Medicine-Cardiovascular Medicine, University of Iowa, Iowa City, IA 52242; ^g^Iowa City Veterans Affairs Health Care System, University of Iowa, Iowa City, IA 52242

**Keywords:** dystroglycan, *O*-mannosylation, matriglycan, cardiac muscle, t-tubule

## Abstract

Maintaining the structure of cardiac membranes and membrane organelles is essential for heart function. A critical cardiac membrane organelle is the transverse tubule system (called the t-tubule system) which is an invagination of the surface membrane. A unique structural characteristic of the cardiac muscle t-tubule system is the extension of the extracellular matrix (ECM) from the surface membrane into the t-tubule lumen. However, the importance of the ECM extending into the cardiac t-tubule lumen is not well understood. Dystroglycan (DG) is an ECM receptor in the surface membrane of many cells, and it is also expressed in t-tubules in cardiac muscle. Extensive posttranslational processing and *O*-glycosylation are required for DG to bind ECM proteins and the binding is mediated by a glycan structure known as matriglycan. Genetic disruption resulting in defective *O*-glycosylation of DG results in muscular dystrophy with cardiorespiratory pathophysiology. Here, we show that DG is essential for maintaining cardiac t-tubule structural integrity. Mice with defects in *O*-glycosylation of DG developed normal t-tubules but were susceptible to stress-induced t-tubule loss or severing that contributed to cardiac dysfunction and disease progression. Finally, we observed similar stress-induced cardiac t-tubule disruption in a cohort of mice that solely lacked matriglycan. Collectively, our data indicate that DG in t-tubules anchors the luminal ECM to the t-tubule membrane via the polysaccharide matriglycan, which is critical to transmitting structural strength of the ECM to the t-tubules and provides resistance to mechanical stress, ultimately preventing disruptions in cardiac t-tubule integrity.

## Significance

Cardiac muscle has a unique membrane organelle called transverse tubule (t-tubule) system which is an invagination of the surface membrane. Preserving the structural integrity of the t-tubule system is necessary for cardiac muscle contraction and, thus, overall heart function. Here, we report that O-glycosylation of dystroglycan (DG) and the formation of matriglycan are essential for maintaining the structural integrity of the cardiac t-tubule system. Importantly, our findings unveil the physiological significance of O-glycosylated DG within cardiac muscle t-tubules, thereby providing evidence of an extracellular matrix-interacting, transmembrane protein that plays a vital role in preserving the structural integrity of t-tubule membranes. Collectively, our findings provide essential advances in elucidating the contribution of cardiac t-tubule disruption in heart disease.

The extracellular matrix (ECM) is critical for development and homeostasis (1). Divided into two regions, the ECM is composed of the basement membrane and the interstitial matrix. In the heart, the basement membrane links directly to the cardiac muscle fiber membrane (i.e., sarcolemma) via transmembrane receptors. Cardiac and skeletal muscle cells contain transverse (t)-tubules, invaginations of the sarcolemma that create a penetrating cellular network. A unique feature of cardiac muscle fibers is the presence of ECM within the t-tubule (transverse tubule) lumen (2–4) (*SI Appendix*, Fig. S1). Moreover, transmembrane receptors have also been identified within cardiac t-tubule membranes (2, 4). While these data suggest a role for the linkage of the t-tubule membrane to the luminal ECM, it is unknown whether the interaction is essential to develop, maintain, or assist in the function of cardiac t-tubules.

**Fig. 1.**
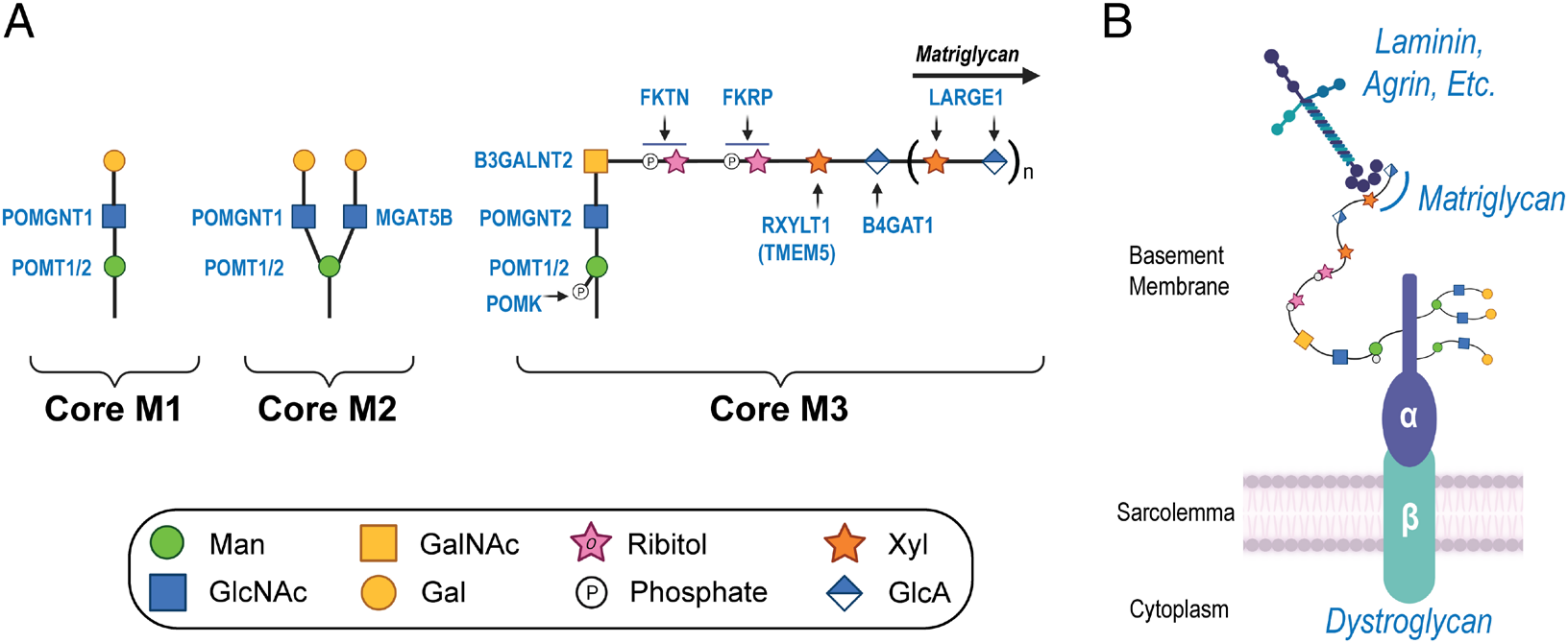
Biosynthesis of core M glycans on α-DG. (*A*) The α-DG *O*-mannosyl glycosylation pathway is illustrated, highlighting the synthesis of core M1, M2, and M3 glycans. (*B*) Illustration depicts *O*-mannosylated DG situated at the sarcolemma and its interaction with ECM ligands. Abbreviations: Man, mannose; GlcNAc, *N*-acetyl-glucosamine; Gal, galactose; GalNAc, *N*-acetyl-galactosamine; Xyl, xylose; GlcA, glucuronic acid; POMT1/2, protein *O*-mannosyltransferases 1 and 2; POMGNT1, protein *O*-linked mannose *N*-acetyl-glucosaminyltransferase 1; POMGNT2, protein *O*-linked mannose *N*-acetyl-glucosaminyltransferase 2; MGAT5B, mannosyl α1,6-glycoprotein β1,6,-*N*-acetyl-glucosaminyltransferase; POMGNT2, protein *O*-linked mannose *N*-acetyl-glucosaminyltransferase 2; B3GALNT2, β1,3-*N*-acetylgalactosaminyltransferase 2; POMK, protein *O*-mannose kinase; FKTN, Fukutin; FKRP, Fukutin-related protein; RXYLT1, ribitol xylosyltransferase 1; TMEM5, transmembrane protein 5; B4GAT1, β1,4-glucuronyltransferase 1; LARGE1, like-acetyl-glucosaminyltransferase 1.

Dystroglycan (DG) is a transmembrane receptor linking the ECM to the cell membrane. Our laboratory has previously identified the presence of DG and matriglycan within cardiac t-tubules (2), although their purpose within these structures remains uncertain. DG is composed of two subunits: The peripheral membrane α-subunit (α-DG) and a transmembrane β-subunit (β-DG). *O*-mannosylation within the mucin-like domain of α-DG results in three subtypes of glycans referred to as cores M1, M2, and M3 (5) (Fig. 1*A*). This process is initiated in the endoplasmic reticulum (ER) by an enzyme complex composed of heterodimerized protein *O*-mannosyltransferases 1 and 2 (POMT1 and POMT2), which catalyzes the transfer of mannose to the hydroxyl oxygen of serine or threonine side chains within the mucin-like domain of α-DG. The core M1 glycan results from addition of β1,2-linked *N*-acetylglucosamine (GlcNAc) to the *O*-mannose by protein *O*-linked mannose *N*-acetyl-glucosaminyltransferase 1. Core M1 glycans can be further extended and can serve as a precursor for core M2 glycans. The core M2 glycan results from adding β1,6-linked GlcNAc to the core M1 glycan through the activity of mannosyl α1,6-glycoprotein β1,6-*N*-acetyl-glucosaminyltransferase (MGAT5B). Several core M2 glycan structures exist. The generation of core M3 glycans begins with the addition of β1,4-linked GlcNAc to the *O*-mannose by POMGNT2. Extension continues with the addition of β1,3-linked *N*-acetyl-galactosamine to the GlcNAc by β1,3-*N*-acetylgalactosaminyltransferase 2. After the trisaccharide is assembled within the ER, protein *O*-mannose kinase phosphorylates the carbon six-position of the *O*-mannose. Further elongation of the phosphorylated core M3 takes place in the Golgi complex. Tandem ribitol-1-phosphate additions are catalyzed by Fukutin (FKTN) and Fukutin-related protein (FKRP). The ribitol is then modified with β1,2-xylose (Xyl) by ribitol xylosyltransferase 1 [RXYLT1; formerly transmembrane protein 5], followed by transfer of β1,4-linked glucuronic acid (GlcA) to the Xyl by β1,4-glucuronyltransferase 1 (B4GAT1). RXYLT1 and B4GAT1 modifications serve as a primer for extension by like-acetyl-glucosaminyltransferase 1 (LARGE1), which then polymerizes an α1,3-Xyl-β1,3-GlcA repeat. The sequential actions of the mentioned glycosyltransferases and kinase yield the heteropolysaccharide known as matriglycan (6). The fully elaborated core M3 glycan structure can functionally bind to laminin globular (LG)-domain-containing ECM proteins such as laminin, agrin, and perlecan (Fig. 1*B*). Although considerable progress has been made in understanding the functional role of the core M3 glycan, the functions of core M1 and M2 glycan structures are not known.

Defective *O*-mannosyl glycan processing results in the complete absence or synthesis of a short form of matriglycan which interferes with DG receptor function (5–8) and underlies a family of diseases known as dystroglycanopathies (9). Cardiac abnormalities have been associated with severe and milder forms of dystroglycanopathies (10, 11), indicating that functional DG is critical for normal heart function. However, our understanding of cardiac disease development and progression due to improper glycosylation of DG is incomplete.

Accumulating evidence shows that disruptions to, and aberrant remodeling of, the cardiac t-tubule network contribute to heart disease (12–21). In general, aberrant remodeling of cardiac t-tubules decreases the amplitude and synchronicity of the systolic Ca^2+^ transient (14, 22), thereby reducing contractile function (14–16, 18, 21, 23, 24). The functional consequences are due to a spectrum of structural changes to the network, including loss or severing of t-tubules and dilated or constricted lumens (20, 21, 25–29). Several proteins have been implicated in t-tubule formation and/or maintenance in cardiac muscle, yet it remains unclear whether linkage to the luminal ECM is required. Here, we show that improper modification of DG increases susceptibility to stress-induced t-tubule disruption and sarcolemma injury, likely due to impaired membrane-to-ECM binding, thus, providing evidence of a functional role for *O*-mannosylated DG within cardiac t-tubules.

## Results

### Core M Glycans on Cardiac Muscle Fiber DG Are Necessary to Prevent Progressive Cardiomyopathy in Mice.

**Fig. 2.**
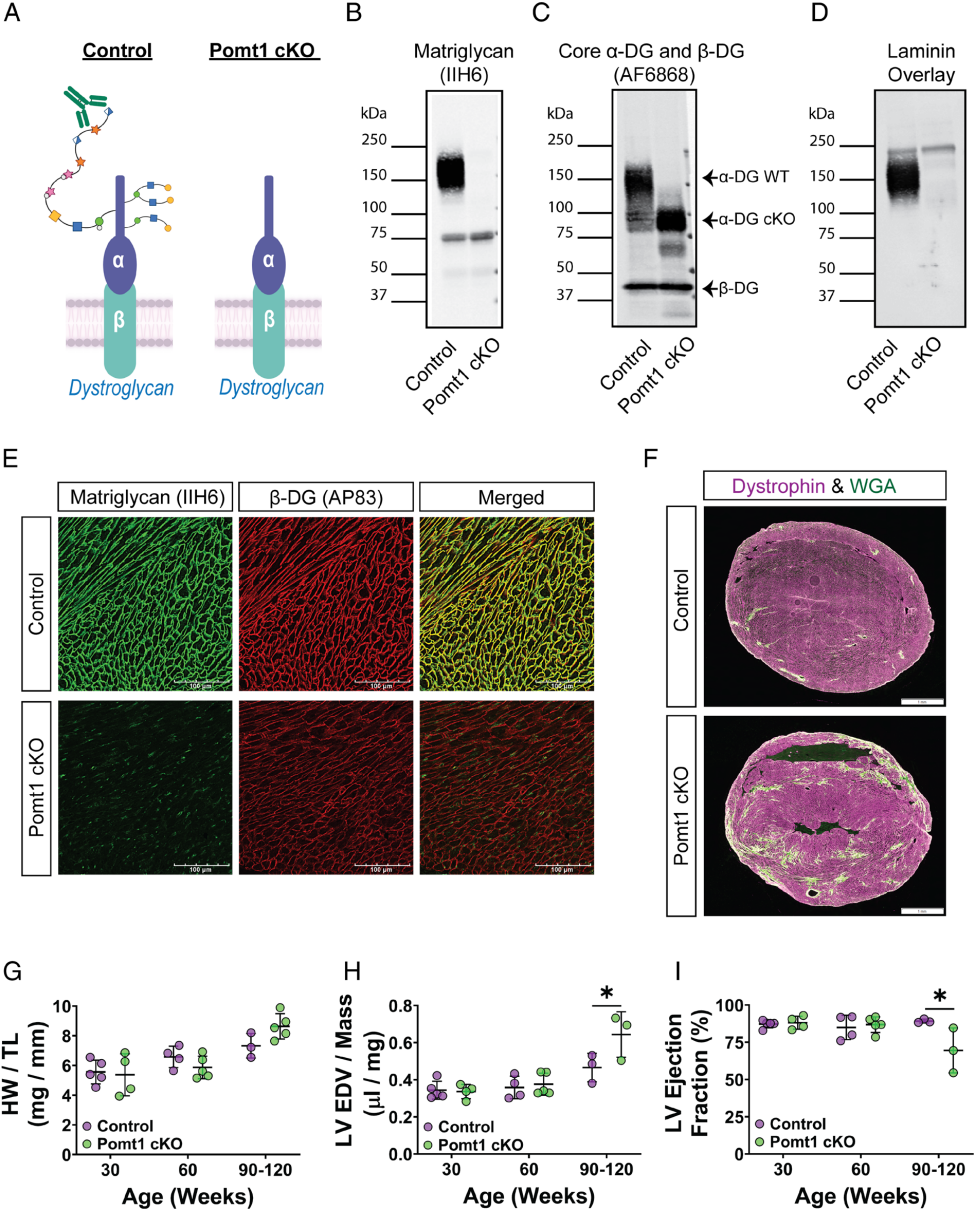
Lack of core M glycans on α-DG in cardiac muscle leads to progressive cardiomyopathy. (*A*) Alpha-DG core M modification in control and Pomt1 cKO. (*B*–*D*) Immunoblots on ventricles to detect *B*, matriglycan; *C*, core α-DG/β-DG; and *D*, laminin binding. Two sets of pooled samples per group (control *n* = 13; cKO *n* = 11). (*E*) Immunofluorescence on ventricles to detect matriglycan and β-DG (*n* = 12 controls; *n* = 12 cKO mice). (Scale bar, 100 μm.) (*F*) Immunofluorescence on ventricles from 120-wk-old mice to detect fibrotic accumulation. (Scale bar, 1 mm.) (*G*) Heart weight per tibial length (HW/TL); (*H*) left ventricular end diastolic volume per left ventricular mass (LV EDV/Mass); and (*I*) LV ejection fraction in 30-, 60-, and 90-120-wk-old mice. Mice of both sexes were utilized. Number of mice for HW/TL and echocardiography: controls = 5; cKO = 4 at 30-wk; controls = 4, cKO = 5 at 60-wk; controls = 3, cKO = 5 for HW/TL at 90 to 120-wk; and controls = 3, cKO = 3 for echocardiography at 90 to 120-wk. *P < 0.05. Unpaired *t* tests with the Holm–Sidak post hoc test performed on age-matched groups. Data expressed as mean ± SD.

Mice with constitutive deletion of *Pomt1* are embryonic lethal due to disruption of Reichert’s membrane during embryogenesis (30). Therefore, to evaluate how the loss of *O*-mannosylated DG in cardiac muscle affects the development and progression of cardiomyopathy, we generated mice in which the *Pomt1* gene was deleted specifically in striated muscle (Pomt1 cKO; *SI Appendix*, Fig. S2 *A* and *B* and Fig. 2*A*). Due to the lack of commercially available or other known antibodies that reliably and effectively detect POMT1 protein in mouse tissues, we indirectly assessed expression of POMT1 by detecting matriglycan.

Wheat-germ agglutinin (WGA)-enriched extracts of cardiac muscle from *Pomt1*^loxP/+^ and *Pomt1*^loxP/loxP^ (control), and *Pomt1*^loxP/loxP^/*MCK*^Cre^ (Pomt1 cKO) mice showed that matriglycan was absent in Pomt1 cKO mice (Fig. 2*B*). However, core α-DG protein was detected at a reduced molecular weight in Pomt1 cKO cardiac muscle (Fig. 2*C*), thus confirming that *O*-mannosylation of α-DG was atypical. Furthermore, we observed broad bands of α-DG-laminin binding in control cardiac muscle, whereas this was greatly reduced in Pomt1 cKO muscle (Fig. 2*D*), indicating a near complete loss of α-DG functional binding in the absence of POMT1. Immunofluorescent analysis revealed that matriglycan was absent in ventricles from Pomt1 cKO mice, while ventricles from controls displayed strong staining for matriglycan along the sarcolemma (Fig. 2*E*). β-DG was observed along the cardiomyofiber sarcolemma in sections from both control and Pomt1 cKO mice (Fig. 2*E*), thus confirming the presence of sarcolemma-localized DG.

To assess the development and progression of cardiac abnormalities due to lack of *O*-mannosylated DG, we examined histological, morphological, and left ventricular (LV) function under baseline conditions at several ages. By 60 wk of age, Pomt1 cKO ventricles begin to show small focal areas of fibrotic accumulation (*SI Appendix*, Fig. S3), although the heart weight-to-tibial length ratio was similar to that of controls (Fig. 2*G*). Between 90 to 120 wk of age, Pomt1 cKO displayed myocardial fibrosis (Fig. 2*F*) along with a nonsignificant increase in heart weight-to-tibial length ratio (Fig. 2*G*). Echocardiographic data showed no impairment in LV function in 30- or 60-wk-old Pomt1 cKO mice compared to age-matched controls (Fig. 2 *H* and *I*). However, by 90 wk of age, LV function in Pomt1 cKO hearts began to indicate progressive dysfunction with an elevated LV end diastolic volume to LV mass ratio (*P* = 0.04), and a reduced LV ejection fraction (*P* = 0.03) compared to age-matched controls.

Together these findings support the conclusion that lack of core M glycans and disruption of DG function is sufficient to cause a mild, progressive cardiomyopathy under nonstressful conditions.

### DG Core M Glycans Offer Resistance Against Injurious Effects Caused by Cardiac Muscle Catecholamine Stress.

**Fig. 3.**
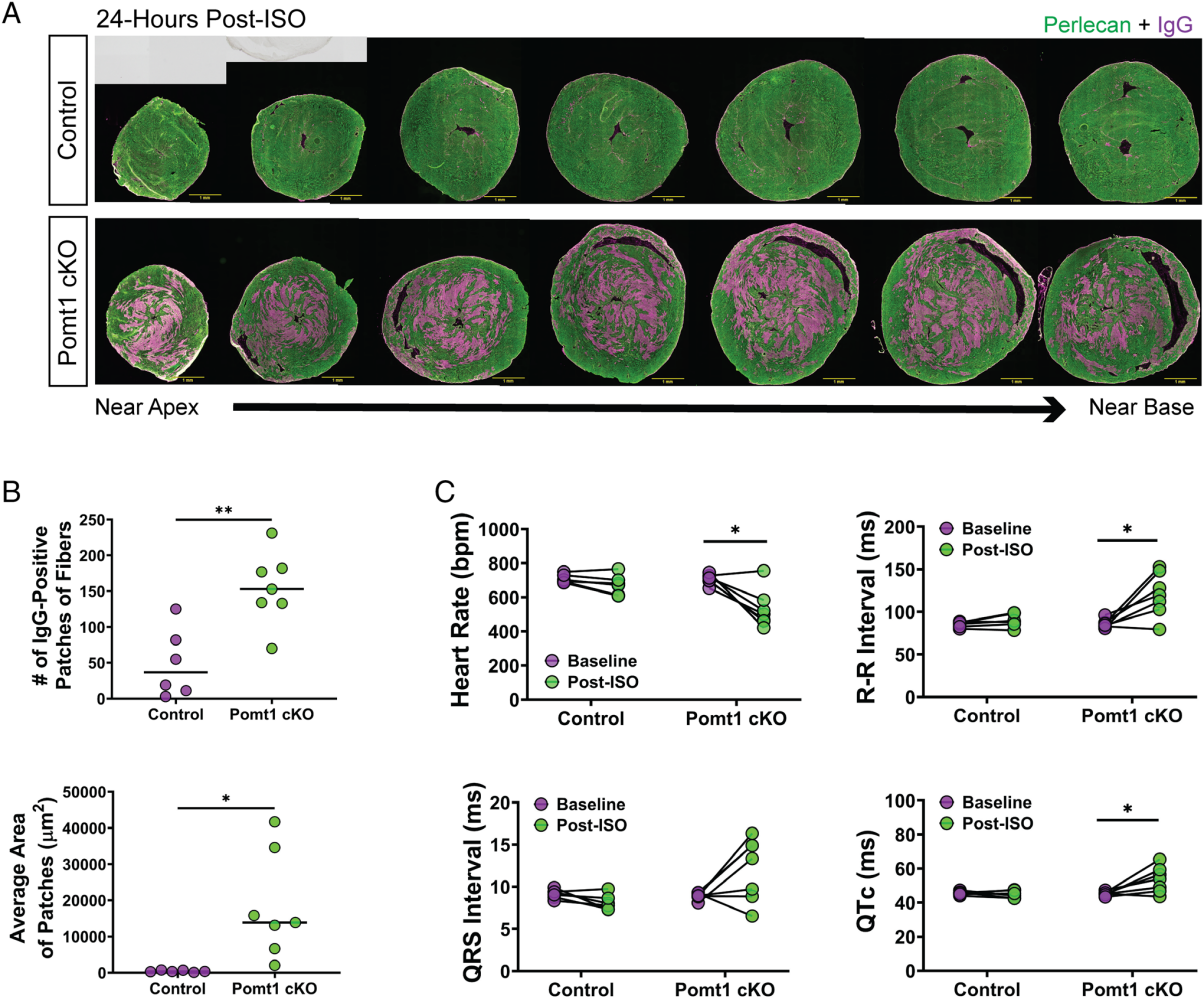
Myocardial stress leads to contractile-induced damage and disrupts normal electrophysiology in Pomt1 cKO mice. ISO (10 mg/kg body weight) was administered to promote an acute bout of increased cardiac workload in control and Pomt1 cKO mice. Mice were killed 24-h post-injection. (*A*) Cardiac cross-sections through the ventricles to assess cardiomyofiber damage as detected by intramyocyte IgG. (Scale bar, 1 mm scale.) (*B*) Quantification of the number of IgG-positive fiber patches (*Upper*) and average area of the patches (*Bottom*) in control and Pomt1 cKO hearts. Image analysis was performed on hearts of mice from both sexes (*n* = 6 controls; *n* = 7 cKO). *P < 0.05; **P = 0.008. Unpaired *t* tests with the Holm–Sidak post hoc test were performed. Data expressed as mean ± SD. (*C*) Electrophysiological analysis of control and Pomt1 cKO mice at baseline and 24-h post-ISO to determine heart rate, R-R interval, QRS interval, and corrected QT intervals (QTc). Experiments were performed with mice of both sexes. Controls, *n* = 6; Pomt1 cKO, *n* = 7 for baseline and post-ISO. *P < 0.05. Paired *t* tests with the Holm–Sidak post hoc test were implemented. Data expressed as mean ± SD.

Due to the progressive nature of cardiac disease often observed in patients with dystroglycanopathies and our Pomt1 cKO mice, we set out to examine the importance of *O*-mannosylated DG in preventing myofiber injury and aberrant remodeling. We assessed myofiber damage at time points prior to detectable morphological or functional aberrations (i.e., between 16 to 36 wk of age) by measuring immunoglobulin G (IgG) uptake, which indicates myofiber membrane damage, 24 h after a β-adrenergic challenge. Control and Pomt1 cKO mice received an intraperitoneal (i.p.) injection of 10 mg/kg body weight of isoproterenol (ISO), a synthetic catecholamine, to induce an increase in heart rate and force of contractility for an acute period (approximately 15 to 30 min). Few IgG-positive cells were detected in control hearts and were mostly limited to individual cardiomyocytes. In stark contrast, Pomt1 cKO appeared ischemic with large patches of IgG-positive myocytes (Fig. 3 *A* and *B* and *SI Appendix*, Fig. S4*A*). Many of the IgG-positive cells in both control and Pomt1 cKO hearts displayed inconsistent immunofluorescent labeling of sarcolemma proteins, including dystrophin, suggesting sarcolemma injury had occurred (*SI Appendix*, Fig. S4*B*). Notably, the β-adrenergic challenge led to the death of some Pomt1 cKO mice, although no control mice expired due to the challenge. To confirm the importance of POMT1 in the susceptibility to cardiomyofiber injury, we overexpressed *Pomt1* in Pomt1 cKO mice using an adeno-associated virus (AAV2/9) construct in which *Pomt1* expression is driven by the *MCK* (muscle creatine kinase) promoter (AAV2/9-MCK-Pomt1). Twenty-four weeks later, mice were subjected to myocardial stress. Levels of matriglycan were restored in both cardiac and skeletal muscle in Pomt1 cKO mice that received AAV2/9-MCK-Pomt1 (*SI Appendix*, Fig. S5*A*). As expected, the forced expression resulting in an increase in POMT1 limited the stress-induced myocardial injury in AAV-treated Pomt1 cKO mice (*SI Appendix*, Fig. S5*B*).

Next, we examined the impact of catecholamine stress on the electrophysiology of the heart. At baseline, control and Pomt1 cKO mice had similar heart rates and sinus rhythm (Fig. 3*C*). Twenty-four hours after receiving ISO, the electrophysiology in the hearts of control mice was seemingly unaffected, whereas Pomt1 cKO mice showed a significantly reduced heart rate, increased R-R intervals, and elevated QTc compared to their baseline values (Fig. 3*C*). Of note, the challenge did result in some Pomt1 cKO mice succumbing to death prior to poststressor testing as well as mice that showed polymorphic ventricular arrhythmia, thus excluding those mice from Fig. 3*C*. Taken together, these findings suggest that prior to the manifestation of cardiac abnormalities, an increased cardiac workload triggers extensive myocardial damage, impairs electrophysiological function, and can even cause sudden death in mice lacking core M glycans on α-DG.

We next examined the influence of cardiac stressors on disease progression. Twenty-four-week-old control and Pomt1 cKO mice received a single i.p. injection of ISO (10 mg/kg body weight). A single stressful event led to death in 3 out of 17 Pomt1 cKO mice, whereas all control mice survived (*SI Appendix*, Fig. S6*A*). Twenty-eight days postchallenge, control hearts displayed a near full recovery (*SI Appendix*, Fig. S6*B*). In contrast, Pomt1 cKO hearts displayed extensive myocardial remodeling that resulted in large fibrotic scars within the myocardium (*SI Appendix*, Fig. S6*B*).

We also sought to evaluate the influence of repeated stressors on disease progression. The first approach involved four challenges (10 mg/kg body weight i.p. ISO) over 4 wk followed by a 28 to 30-d recovery period (*SI Appendix*, Fig. S6*C*). Challenged Pomt1 cKO mice either succumbed to the stressors (*SI Appendix*, Fig. S6*D*) or exhibited impaired LV function (*SI Appendix*, Fig. S6 *E* and *F*). Our second approach involved 10 daily challenges with a lower dose of ISO (2.5 mg/kg body weight i.p.) (*SI Appendix*, Fig. S7*A*). Stressed Pomt1 cKO mice either died (*SI Appendix*, Fig. S7*B*) or their ventricles displayed signs of damage and aberrant remodeling (*SI Appendix*, Fig. S7 *C* and *D*). The challenges had minimal impact on control mice.

Overall, these results indicate that acute and repeated cardiac stressors of low-to-moderate-intensity cause lasting myocardial damage, and in some cases, death of Pomt1 cKO mice.

### Development of the T-tubule Network Does Not Require DG Modified with Core M Glycans.

**Fig. 4.**
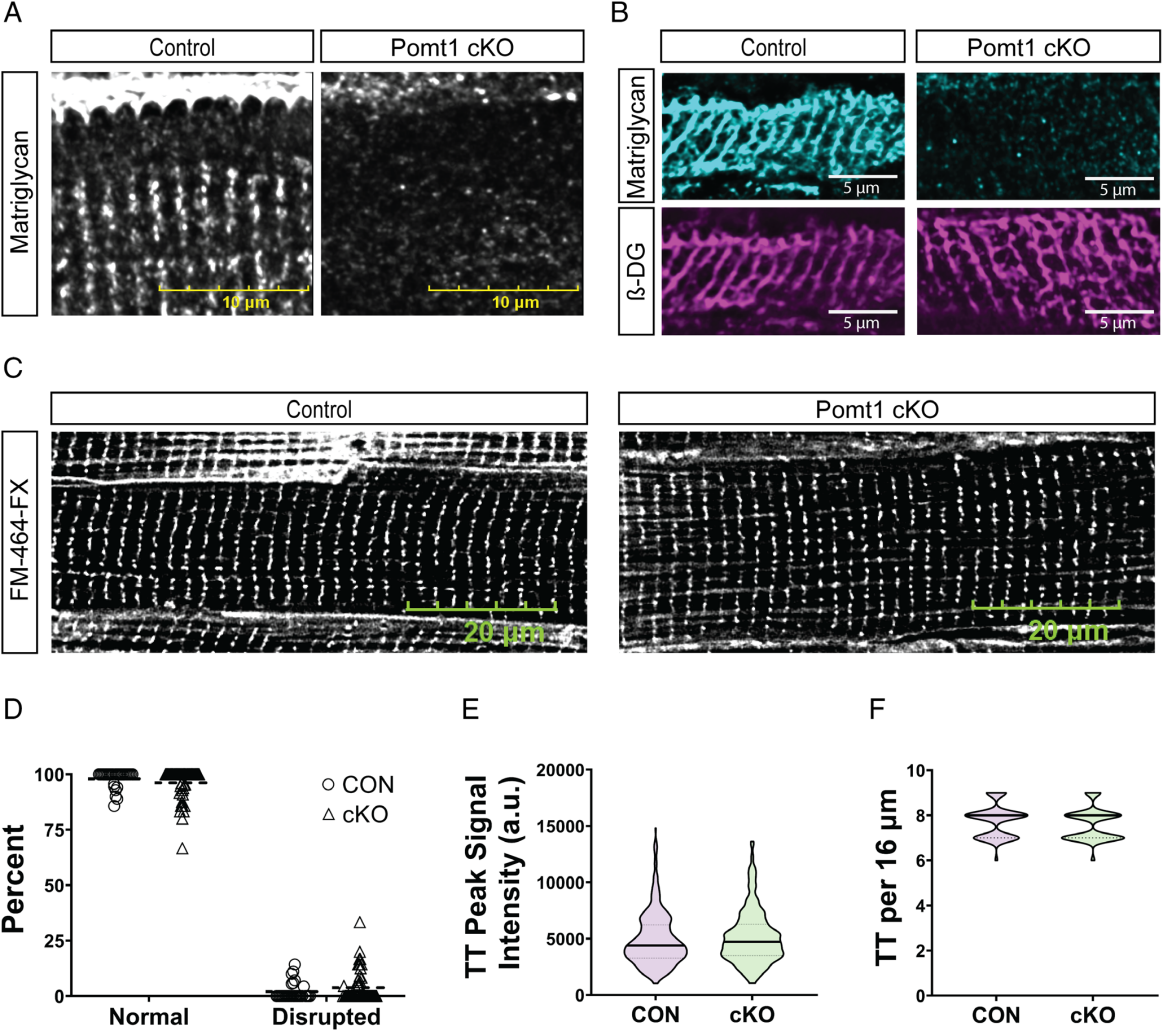
The t-tubule network develops in the absence of *O*-mannosylated α-DG. (*A* and *B*) Immunofluorescence of cryosections of ventricles from control and Pomt1 cKO mice to detect matriglycan (*A* and *B*) and β-DG (*B*). Ten µm cryosections were used in *A*. (Scale bar, 10 µm.) Sixteen µm cryosections were used in *B*. (Scale bar, 5 µm.) (*C*) FM 464-FX fluorescence of whole left ventricles to detect plasma membranes, including t-tubule membranes. (Scale bar, 20 µm.) (*D*) Quantification of the percent of myofibers with normal or disrupted t-tubule staining within a 90× magnification field. (*E*) Line scan analysis to determine the peak signal intensity of FM 464-FX labeled t-tubules (TT). (*F*) Number of t-tubules observed within 16 µm regions. Image analysis was performed on hearts of mice from both sexes (*n* = 4 controls; *n* = 5 cKO). Unpaired *t* tests with the Holm–Sidak post hoc test were performed. Data are expressed as mean ± SD.

In large mammals, such as sheep, DG and matriglycan are localized to the sarcolemma and the t-tubule membranes within cardiomyofibers (2). Additionally, we provide evidence of t-tubule membrane matriglycan and t-tubule lumen basement membrane within human cardiac muscle fibers (*SI Appendix*, Fig. S8). Immunofluorescence of control mouse ventricles and confocal microscopy confirmed the presence of the basement membrane within cardiac t-tubule lumens (Fig. 4 *A* and *B* and *SI Appendix*, Figs. S1*A* and S9*A*). This feature is unique to cardiac muscle as ECM is not detected within the t-tubule lumen in skeletal muscle (*SI Appendix*, Fig. S1*B*). We also detected junctional complex proteins, ryanodine receptor 2 and junctophilin 2 (JP2), and t-tubule membrane proteins caveolin-3 (*SI Appendix*, Fig. S9*B*), β-DG (Fig. 4*B*), and matriglycan (Fig. 4 *A* and *B*). We observed the same junctional complex, t-tubule lumen, and membrane markers in cryosections from Pomt1 cKO ventricles, except for matriglycan (Fig. 4 *A* and *B* and *SI Appendix*, Fig. S9 *A* and *B*). We also found similar protein amounts of the dyadic DHPRα2 and the t-tubule membrane-localized BIN1 in control and Pomt1 cKO WGA-enriched cardiac muscle (*SI Appendix*, Fig. S9 *C* and *D*). Next, we examined t-tubule alignment in ventricular muscle. Confocal imaging of whole ventricles labeled with FM 464-FX, a plasma membrane marker that labels t-tubules, revealed that deletion of POMT1 did not alter t-tubule organization as determined by control-like appearance (Fig. 4 *C* and *D* and *SI Appendix*, Fig. S10), t-tubule fluorescent intensity (Fig. 4*E*), and number of t-tubules within a region of interest (Fig. 4*F*). Collectively, these findings indicate that core M glycans are not required for t-tubule maturation, including membrane invagination and alignment to the junctional sarcoplasmic reticulum in mice.

### Core M Glycans Are Essential to Preserving T-tubule Structure During Cardiac Stress.

**Fig. 5.**
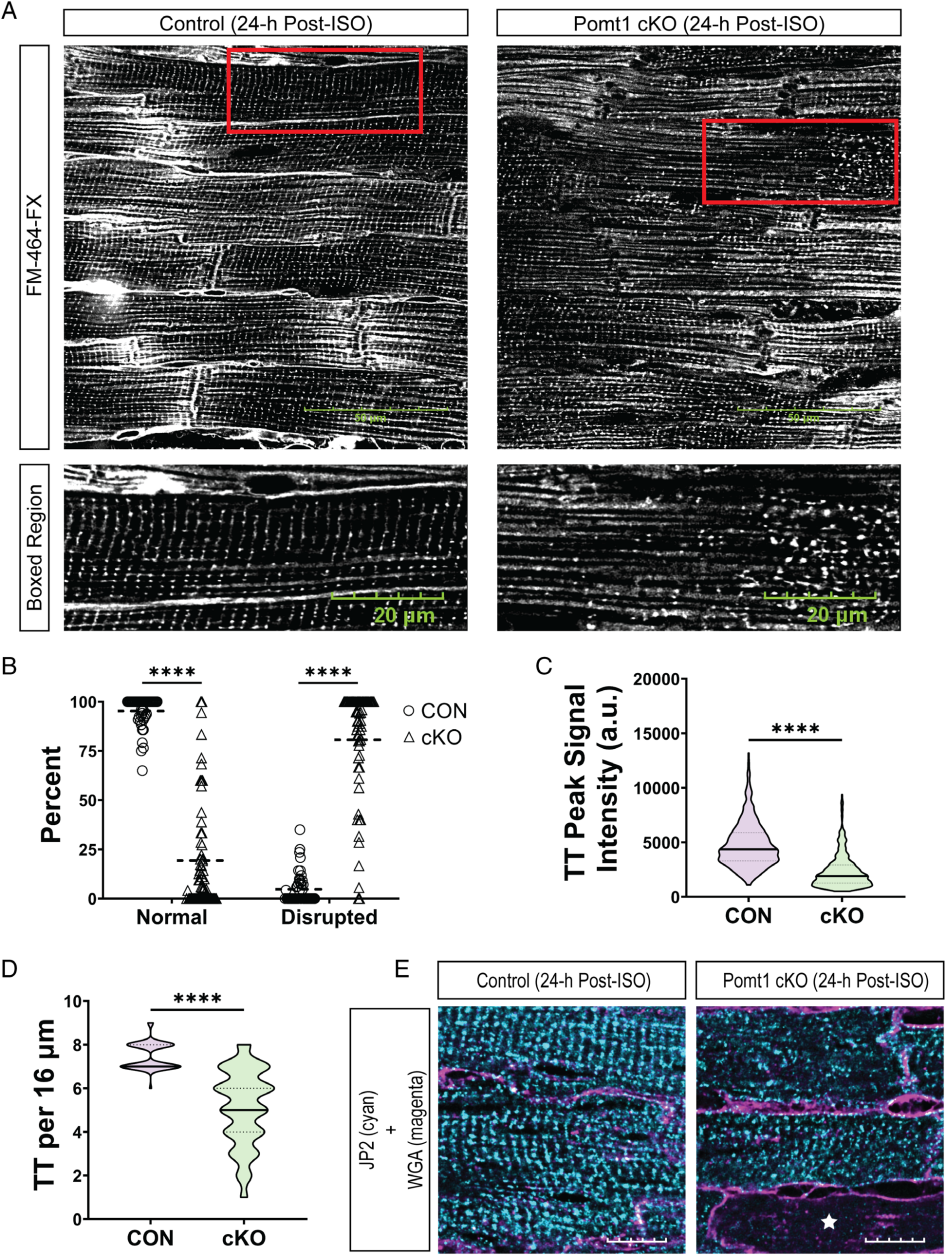
Pomt1 cKO ventricular myocytes exhibit detubulation and disruption of the cardiac dyad in response to contractile stress. (*A*) Labeling of control and Pomt1 cKO whole left ventricles stained with FM 464-FX 24-h after a single bolus of ISO (10 mg/kg body weight). (Scale bar, 50 µm.) *Bottom* images show the boxed region (red rectangular box) from the *Upper* images. (Scale bar, 20 µm.) (*B*) Percentage of myofibers that showed either normal or disrupted patterns of t-tubules. (*C*) Line scan analysis to determine the peak signal intensity of FM 464-FX labeled t-tubules (TT). (*D*) Number of t-tubules observed within a 16 µm region. Image analysis was performed on hearts of mice from both sexes (*n* = 4 controls; *n* = 5 cKO). Unpaired *t* tests with the Holm–Sidak post hoc test were performed. Data expressed as mean ± SD. ****P < 0.0001. (*E*) Immunofluorescence to detect JP2 and WGA-AlexaFluor 594 in hearts from mice subjected to a single bolus of ISO. (Scale bar, 10 µm.) The white star in the Pomt1 cKO image indicates a cardiomyofiber that lacked JP2 immunodetection.

To determine if the absence of *O*-mannosylation disrupts t-tubule structural integrity in the context of cardiac stress, we labeled freshly harvested, LV from control and Pomt1 cKO hearts with the FM 464-FX membrane dye and assessed the t-tubule network. Twenty-four hours after a β-adrenergic challenge, control cardiomyofibers retained baseline t-tubule orientation (Fig. 5*A*). However, t-tubules in stressed Pomt1 cKO cardiomyofibers had a disrupted appearance (Fig. 5 *A* and *B*), reduced t-tubule signal intensity (Fig. 5*C*), and were lost or fragmented (Fig. 5*D*). Furthermore, we provide evidence that loss and severing of t-tubules occurs in the absence of catastrophic plasma membrane damage in Pomt1 cKO cardiac muscle fibers (Fig. 5*E* and *SI Appendix*, Fig. S11). Catastrophic plasma membrane damage led to the release or degradation of JP2 (Fig. 5*E*) and was also evident by infiltration of FM 464-FX into the myofibers (*SI Appendix*, Fig. S11). These data suggest that *O-*mannosylated DG is critical for cardiac t-tubule structural integrity. Our data indicate that an acute stressor causes t-tubule fragmentation and severing within cardiomyofibers lacking core M glycans.

### Matriglycan Is Critical for Cardiac Muscle T-tubule Membrane Stability.

To address whether the core M3 glycan modification of DG is essential for maintaining myocardial membrane integrity, we generated mice in which the *Large1* gene was deleted in striated muscle (Large1 cKO; *SI Appendix*, Fig. S12*A*). The LARGE1 protein is the final glycosyltransferase involved in formation of core M3 glycan structures and responsible for generation of matriglycan (*SI Appendix*, Figs. S12*B* and S13*A*). WGA-enriched extracts of cardiac muscle from *Large* ^loxP/+^ and *Large1*^loxP/loxP^ (control) and *Large1*^loxP/loxP^/*MCK*^ Cre^ (Large1 cKO) mice showed that matriglycan was absent in Large1 cKO cardiac muscle (*SI Appendix*, Fig. S13*B*). β-DG was present in Large1 cKO cardiac muscle (*SI Appendix*, Fig. S13*C*), thereby confirming the presence of DG. We observed α-DG-laminin binding in control cardiac muscle, while binding was reduced in Large1 cKO cardiac muscle (*SI Appendix*, Fig. S13*D*), confirming a loss of α-DG functional binding in the absence of LARGE1. Immunofluorescent analysis revealed that matriglycan was absent from Large1 cKO ventricles, while control ventricles displayed sarcolemmal matriglycan (*SI Appendix*, Fig. S13*E*).

Next, we assessed the importance of matriglycan in preventing myocardial injury. A single β-adrenergic challenge (10 mg/kg body weight i.p. ISO) was given to Control and Large1 cKO mice. All four control mice survived the challenge, whereas only three of four Large1 cKO mice survived. Hearts were harvested from surviving mice 24 h after the mice began the challenge. Consistent with observations described in Fig. 3 *A* and *B*, control ventricles displayed few IgG-positive myocytes (*SI Appendix*, Fig. S13*F*), while Large1 cKO ventricles exhibited sprawling patches of IgG-positive cardiomyocytes (*SI Appendix*, Fig. S13*F*). Acute stress also led to disrupted t-tubule appearance (*SI Appendix*, Fig. S14 *A*–*H*), reduced t-tubule fluorescent signal intensity (*SI Appendix*, Fig. S14 *C* and *G*), and t-tubule loss or fragmentation (*SI Appendix*, Fig. S14 *D* and *H*) in Large1 cKO muscle, whereas control fibers retained baseline t-tubule orientation (*SI Appendix*, Fig. S14 *A*–*H*). These data indicate that matriglycan is required in cardiac t-tubule membranes to withstand stress.

Collectively, our findings reveal that matriglycan is required for the integrity of cardiac membrane organelles, and that even in the absence of catastrophic sarcolemma failure, t-tubules are still vulnerable to stress-induced disturbance. Thus, the membrane disruptions likely trigger or at least contribute to the acute failure and progressive cardiac disease observed in preclinical research models and dystroglycanopathy patients.

## Discussion

Our data provide evidence that the membrane-to-ECM link established by the interaction of matriglycan with extracellular ligands is crucial for providing structural integrity to the t-tubule membrane and sarcolemma, and consequently for maintaining cardiomyofiber function and survival. Due to the similarities in cardiac membrane fragility observed in the Pomt1 cKO and Large1 cKO hearts, our data indicate that the core M3 glycan and matriglycan are essential in providing a stabilizing link between the cardiac muscle membranes and ECM. Furthermore, the present data suggest it is unlikely that core M1 and M2 glycans contribute to the stabilization of cardiac muscle membrane organelles. Overall, our results indicate that the linkage established by matriglycan is necessary for limiting stress-induced membrane damage and suggests that myocardial stress is a driver of disease development in dystroglycanopathies.

Importantly, we show that matriglycan is essential for preserving cardiac t-tubule structural integrity, the absence of which compromises t-tubule networks and leaves them susceptible to stress-induced disruption. Although several proteins have been implicated in the morphogenesis and/or maintenance of cardiac t-tubules, including caveolin-3 (31), JP2 (32, 33), BIN1 (34–37), and MG53 (38), no studies have addressed the concept of a membrane-bound ECM receptor that stabilizes t-tubule membranes and secures the integrity of the network. Microtubule derangements have been implicated as the cause of t-tubule disruption in cardiomyocytes from dystrophin-deficient *mdx* mice (39), an effect that stems from the lack of subsarcolemmal dystrophin-microtubule interaction. However, dystroglycanopathy mouse models lack DG-ECM linkage while retaining dystrophin (40) and, therefore, are capable of anchoring microtubules to dystrophin. Thus, our work provides evidence that matriglycan is essential for the maintenance of t-tubule structural integrity during periods of cardiac stress. Notably, a recent study found that isolated cardiomyocytes from *Fukutin* (*Fktn*) cKO mice displayed impaired contractile properties and disrupted excitation–contraction (E-C) coupling (41). Moreover, our present data show that muscle excitability is altered in stressed Pomt1 cKO hearts. Collectively, this suggests a potential mechanism where cardiac muscle cells that lack matriglycan are vulnerable to t-tubule disruption and remodeling, which can disturb E-C coupling and myocyte contraction. Therefore, we propose that t-tubule disruption and aberrant remodeling is a contributing factor in dystroglycanopathy-related cardiac disease.

Our results from a preclinical mouse model of dystroglycanopathy, describe a progressive cardiac disease in sedentary Pomt1 cKO mice, however prior to overt disease development, contractile stress can instigate cardiomyofiber injury and dysfunction. The progressive disease observed in Pomt1 cKO mice is in agreement with findings from recent studies that used mouse models with mutations in *Large1* (42), *Fktn* (41), and *Fkrp* (43), all of which display impaired or complete absence of matriglycan formation. Our observations of large patches of injured cardiomyocytes in younger Pomt1 cKO and Large1 cKO mice following mild cardiac stressors suggest that the accumulation of myocardial injury is likely a driver of disease onset and progression in the matriglycan-deficient heart.

In summary, our data reveal a previously unknown role for matriglycan as a structural stabilizer within cardiac t-tubules. Our findings indicate that cardiac t-tubule DG mediates anchoring of the luminal ECM to the t-tubule membrane via matriglycan and this linkage transfers the structural strength of the ECM to the t-tubule. These findings provide an essential advance in elucidating the contribution of cardiac t-tubule disruption in heart disease.

## Materials and Methods

### Animals.

Animal care, ethical usage, and procedures were performed in strict accordance with protocols approved by the NIH and the Institutional Animal Care Use and Committee. Mice were socially housed (unless single housing was required), under specific-pathogen-free conditions in an Association for Assessment and Accreditation of Laboratory Animal Care-accredited animal facility. Mouse housing conditions were as specified in the Guide for the Care and Use of Laboratory Animals (National Research Council). A reverse 12 h/12 h light/dark cycle was used, and in vivo mouse assessments only took place during the dark cycle. Standard rodent chow (Harlan Laboratories) and water were available ad libitum. *Pomt1*^loxP/loxP^ and *Large1*^loxP/loxP^ mice were used as controls. When available, littermates were used as controls. Both male and female mice were used. Within each experiment, mice were age-matched and sex-matched when possible. Group designations (randomization) were assigned based on identification numbers and genotype information before the experimenter observed the mice to exclude any bias based on mouse phenotype. Animal usage and data reporting were in accordance with the Animal Research: Reporting of In Vivo Experiments guidelines.

### Generation of *Pomt1* Conditional Knockout Mice.

*Pomt1*-targeted mouse embryonic stem (ES) cells were obtained from the European Conditional Mouse Mutagenesis Program and injected into C57BL/6 blastocysts to generate chimeras. The resulting chimeric mice were mated with C57BL/6 wild-type mice, and their progeny were screened for transmission of the *Pomt1*-targeted allele. Mating between a mouse bearing a *Pomt1*-targeted gene and a mouse bearing the flippase recombinase transgene (FLP) resulted in progeny that were heterozygous for the *Pomt1*-floxed allele (*Pomt1*^loxP/+^). These mice were interbred to produce homozygous floxed *Pomt1* (*Pomt1*^loxP/loxP^) mice. These mice were subsequently bred onto a C57BL/6J background for six generations.

To generate a striated muscle-specific *Pomt1* conditional cKO mouse model, we designed a breeding strategy based on the Cre/LoxP system aimed at obtaining target mice carrying homozygous *Pomt1* floxed alleles and the *MCK*-Cre transgene (*Pomt1*^loxP/loxP^/*MCK*^Cre^). Cre recombinase activity is driven by the promoter of the *MCK* gene. Cre expression under the *MCK* promoter directs recombination in differentiating striated muscle cells. *MCK* becomes active with differentiation and is first detected in skeletal and cardiac muscles at embryonic day 17 and reaches maximal sustained expression in striated muscle fibers by postnatal day 10 (44). As *MCK* is not expressed in stem cells, myoblasts, or satellite cells, development and regeneration are spared in *MCK*^Cre^ KO models. Transgenic *MCK*^Cre^ mice [B6.FVB(129S4)-Tg(Ckmm-cre)5Khn/J; stock no. 006475] were purchased from Jackson Laboratory.

*Pomt1*^loxP/loxP^ mice were crossed with mice expressing *MCK*^Cre^ to produce mice heterozygous for the *Pomt1* floxed allele and the *MCK*^Cre^ transgene (*Pomt1*^loxP/+^/*MCK*^Cre^). To achieve *Pomt1*^loxP/loxP^/*MCK*^Cre^ target mice, male *Pomt1*^loxP/+^/*MCK*^Cre^ mice were bred with female *Pomt1*^loxP/loxP^ mice. All mice were subjected to genotyping either by PCR analysis or outsourced to Transnetyx (Cordova, TN). In striated muscle tissues, exons 3 and 4 of *Pomt1* were expected to be deleted in both *Pomt1* alleles by Cre-driven recombination, thus generating *Pomt1* null alleles. For the assessment of intragenic *Pomt1* deletion in mouse striated muscle genomic DNA, muscle was extracted, and DNA was isolated. Primers, *Pomt1*-Forward primer 5′-CCA CCC AGC ACT TAA CCT TTT A-3′ and *Pomt1*-Reverse primer 5′-ACT GTA TAT GCC TGG CCA CTG T-3′ yielded a 223 bp product for wild-type and a 203 bp band for *Pomt1*-floxed. *MCK*^Cre^ transgene expression was performed as instructed by Jackson Laboratory using the transgene forward primer (oIMR6754) 5′-TAA GTC TGA ACC CGG TCT GC-3′ and transgene reverse primer (oIMR1085) 5′-GTG AAA CAG CAT TGC TGT CAC TT-3′ to yield a 450 bp product for the transgene.

### Generation of *Large1* Conditional Knockout Mice.

An ES cell line with a targeted mutation in *Large1* was obtained from the Knockout Mouse Project Repository at the University of California Davis and injected into C57BL/6 blastocysts to generate chimeric mice. The resulting chimeras were mated with C57BL/6 wild-type mice, and their progeny were screened for transmission of the *Large1*-targeted allele. Mating between a mouse bearing a *Large1*-targeted gene and a mouse bearing the flippase recombinase transgene (FLP) resulted in progeny that were heterozygous for the *Large1*-floxed allele (*Large1*^loxP/+^). The resulting mice were interbred to produce homozygous floxed *Large1* (*Large1*^loxP/loxP^) mice. Finally, the progeny were subsequently bred onto a C57BL/6J background for six generations.

*Large1*^loxP/loxP^ mice were crossed with *MCK*^Cre^ mice to produce mice heterozygous for the *Large1* floxed allele and the *MCK*^Cre^ transgene (*Large1*^loxP/+^/*MCK*^Cre^). Male *Large1*^loxP/+^/*MCK*^Cre^ mice were bred with female *Large1*^loxP/loxP^ mice to achieve *Large1*^loxP/loxP^/*MCK*^Cre^ mice. Every mouse was subjected to genotyping either by PCR analysis or outsourced to Transnetyx. In striated muscle tissues, exon 3 of *Large1* was expected to be deleted in both *Large1* alleles by Cre-driven recombination, thus generating *Large1* null alleles. For the assessment of intragenic *Large1* deletion in mouse striated muscle genomic DNA, muscle was extracted, and DNA was isolated. Primers, *Large1* – Forward primer 5′-TGG CAT TGT GGC AGG TAA CAG-3′ and *Large1* – Reverse primer 5′-TCC ACA CAT GGT ATG TAC TCA CT-3′ yielded a 383 bp product for wild-type and a 444 bp band for *Large1*-floxed.

### Mouse Phenotyping.

To evaluate the cardiovascular health of experimental mice, electrocardiogram recordings and echocardiograph recordings were used to assess the morphology and functionality of the heart.

### Electrocardiography (ECG) Recording.

The ECG experimental protocol was performed in line with the procedures described in Spielmann et al. (45) except where stated. Briefly, mice were permitted to acclimate to the procedure room for 15 min and then were acclimated on the ECG recording platform for 10 min prior to measurement. Electrocardiograms were recorded at the same time of day to eliminate circadian influences. A disposable recording tower with a lead plate (Mouse Specifics, Inc.) embedded into the floor of the recording platform and spaced to provide contact between the electrodes and the paws of the mouse, provided an ECG signal equivalent to that of Einthoven lead II. ECG recordings in which a minimum of 15 ECG beats could be included in the analysis were chosen. Data were analyzed using standard protocols for ECG signal analysis by EzCG Signal Analysis Software (Mouse Specifics, Inc.). EzCG software employs a peak-detection algorithm to detect the peak of R-waves and calculate heart rate. The software plots its interpretation of P, Q, R, S, and T for each heartbeat so that heart rate, QRS duration, PQ interval, PR interval, QT interval, and ST interval are automatically measured and reported. Furthermore, each trace was examined by the experimenter for clear peak identification prior to accepting the automatic reporting and calculations. If R peaks were not chosen correctly, manual corrections were made by the experimenter. Noise and motion artifacts were automatically rejected by EzCG software. Heart rate variability was calculated as the mean of the differences between sequential heart rates for the complete set of ECG signals. Rate correction of QT intervals (QTc) was accomplished by applying the equation recommended by Mitchell et al. (46).

### Echocardiography.

For transthoracic echocardiography recordings, mice were lightly sedated with 100 µL of 0.1 mg midazolam administered via subcutaneous injection. Fur was removed from the chest of the mouse with either Nair (Church & Dwight) or electric clippers. Mice were placed in the supine position in the palm of the hand, gently restrained by the nape, and with their tail held between the last two fingers. Prewarmed ultrasound gel was applied to the imaging target area of the chest. Images of the heart were obtained via a 30-MHz linear array transducer coupled to a Vevo 2100 Imager (FUJIFILM VisualSonics, Bothell, WA). Transthoracic echocardiography recordings of the short and long axis modes were obtained at a frame rate between 180 to 250 Hz. Once imaging was complete, the mouse was returned to its cage and allowed to recover. Cardiac image analysis was blindly performed by a trained sonographer using Vevo 2100 analytical software (v.1.5; VisualSonics). From the short axis view, endocardial and epicardial borders were traced during diastole and systole. Left ventricle length was measured from the endocardial and epicardial borders to the outflow tract during diastole and systole. Finally, LV mass and ejection fraction were calculated via the biplane area-length method.

### Antibodies.

The following primary antibodies have been described previously and were obtained from the listed sources: IIH6 monoclonal antibody (47) to detect matriglycan (Campbell Laboratory; Developmental Studies Hybridoma Bank, University of Iowa; RRID:AB_2617216); affinity purified β-DG rabbit polyclonal AP83 (48) (Campbell Laboratory); AF6868 rabbit polyclonal antibody to detect core αDG and βDG proteins (Campbell Laboratory; R and D Systems, Minneapolis, MN; RRID:AB_10891298); DHPR rabbit polyclonal antibody (48) to alpha2 subunit (Campbell Laboratory); polyclonal anti-laminin (L9393; Sigma-Aldrich, St. Louis, MO); monoclonal anti-perlecan (A7L6; Invitrogen, Waltham, MA); polyclonal JPH2 antibody (40 to 5,300; Invitrogen); monoclonal anti-BIN1 (EPR13463-25; Abcam); and polyclonal anti-caveloin-3 (ab2912; Abcam). Secondary antibodies conjugated to Alexa Fluor 488, Alexa Fluor 555, Alexa Fluor 594, and Alexa Fluor 647 were purchased from Invitrogen.

### Histology and Immunofluorescence.

Mice were killed by cervical dislocation and hearts were immediately harvested. Greater vessels were trimmed, blood was removed from the chambers by wicking, and the wet heart weight was obtained. Hearts were embedded in freezing medium (Tissue-Tek O.C.T. compound; Sakura FineTek; Torrance, CA, USA) and immediately snap frozen in liquid nitrogen-cooled isopentane (2-methylbutane). Ten μm sections were cut with a cryostat (Leica CM3050S Research Cryostat; Amsterdam, the Netherlands) set at −20 °C. Cryosections were processed for hematoxylin and eosin staining according to standard protocols (47). Picrosirius red (0.1%) & Fast Green (0.1%) staining was performed on heart cryosections to evaluate fibrotic accumulation (49). For immunofluorescence, cryosections were blocked with Background Buster (NB306; Innovex Biosciences), incubated in primary antibodies overnight, washed in PBS, followed by incubation in secondary antibodies (1:500), washed in phosphate-buffered saline (PBS), and cover-slipped with mounting medium containing the nuclear marker DAPI (ProLong Gold Antifade Mountant with DAPI; Invitrogen). In select cases, Alexa Fluor-conjugated WGA (1:250; Invitrogen) to detect ECM accumulation and anti-mouse IgG (1:250; Invitrogen) used to detect myofiber damage via immune-cell infiltration were added along with secondary antibodies. Digital images were acquired with either the VS120-S5-FL slide scanner microscope (Olympus Corporation, Tokyo, Japan) or the FLUOVIEW FV3000 confocal laser scanning microscope (Olympus). Quantitative analysis was performed using VS-Desktop software (Olympus) and cellSens analysis software (Olympus).

Mouse hearts were rapidly removed and placed in a dissection dish where it was then repeatedly washed in a modified Tyrode buffer (137 mM NaCl; 5 mM KCl; 1 mM NaH_2_PO_4_; 24 mM NaHCO_3_; 11 mM glucose) until spontaneous beating slowed or ceased. The heart was then washed in the modified Tyrode solution containing 20 mmol/L of 2,3-butanedione monoxime to minimize contractures after cutting. Atrial tissue was excised, followed by careful excision of the ventricles and septum. LV tissue was washed in the modified Tyrode solution three times for five min each. Next, LV tissue incubated in either FM 4-64FX lipophilic dye (10 µg/mL in modified Tyrode; F34653; Thermo Fisher Scientific) for 20 min or WGA conjugated with Alexa Fluor 488 (1:250 in modified Tyrode; Invitrogen) for 1 h at room temperature in a dark chamber. The tissue was fixed with 4% paraformaldehyde for 10 min followed by five washes in PBS. Samples were then mounted in Permafluor mountant (TA-030-FM; Thermo Fisher Scientific) in chambers made with imaging spacers (654006; Grace Bio-Labs) that were stacked and attached to a glass microscope slide. Immediately after mounting, images were acquired using the 60× objective on an Olympus FLUOVIEW FV3000 confocal laser scanning microscope. Only fibers from the outer edge of the myocardium were imaged. Maximum intensity Z-stacks were reconstructed with the FV31S (Olympus) software and deconvoluted with cellSens Dimension (Olympus). T-tubule fluorescence intensity profile and peak intensity analysis was performed with cellSens software. Briefly, same-sized regions of interest (16 µm width × 2.5 µm height) were placed in two locations within each myofiber. Region of interest rectangular boxes were placed at least 2 µm away from the sarcolemma, intracellular perinuclear areas, and intercalated disks in order to avoid signal interference. Box placement was chosen to include representative t-tubule patterns for each myofiber. Background signal was subtracted from each region of interest when determining peak fluorescent intensity. Peak intensity and t-tubule count were averaged across the two regions of interest per myofiber.

Human heart tissue cryosections were purchased from OriGene (CS616190; CS616185; Rockville, Maryland, USA). Five μm frozen tissue sections were mounted onto a glass slide and shipped on dry ice to the University of Iowa. Slides were subsequently stored at −80 °C. Immunofluorescence was performed in the same fashion as that of the ventricular mouse cryosections.

### Glycoprotein Enrichment, Immunoblotting, and Ligand Overlay.

Heart (5 to 6 pooled hearts per group) and skeletal muscle tissue was minced and then placed in Tris-buffered saline (TBS) containing 1% Triton-X-100 and protease inhibitors for processing (50). The solubilized fraction was combined with a WGA-agarose bead slurry (Vector Laboratories, Burlingame, CA) and incubated overnight at 4 °C with rotation. Pellets formed from the beads and were subsequently washed three times in 0.1% Triton X-100/TBS (48). The beads were eluted with 5× Laemmli sample buffer (LSB) at 99 °C for 10 min. A final concentration of 1.11 mg muscle tissue per μL beads and LSB was achieved. Samples were separated by 3 to 15% sodium dodecyl-sulfate polyacrylamide gel electrophoresis (SDS-PAGE) and transferred to polyvinylidene fluoride (PVDF)-FL membranes. The membranes were incubated with primary antibodies followed by the appropriate infrared (IR) dye-conjugated secondary antibodies.

Laminin overlay assays were performed as previously described (50). Briefly, PVDF-FL membranes were blocked in laminin binding buffer (10 mM triethanolamine, 140 mM NaCl, 1 mM MgCl_2_, 1 mM CaCl_2_, pH 7.6) containing 5% milk followed by incubation with mouse Engelbreth–Holm–Swarm laminin (ThermoFisher Scientific, 23017015) overnight at 4 °C in laminin binding buffer containing 3% bovine serum albumin with 2 mM CaCl_2_. Membranes were then washed and incubated with anti-laminin (L9393; Sigma-Aldrich, 1:100 or 1,000) followed by IRDye 800CW dye-conjugated donkey anti-rabbit IgG (LI-COR, 926-32213; 1:2,500).

Immunoblots and laminin overlays were scanned using the Odyssey IR imaging system (LI-COR Bioscience) and images were subsequently captured with Odyssey image analysis software (LI-COR Bioscience).

### β-Adrenergic Challenge.

β-adrenergic stimulation was induced by i.p. injection of ISO under light isoflurane anesthesia (induction at 3.5%). (-)-ISO hydrochloride (I6504; Sigma-Aldrich) was dissolved in saline to a concentration of 6 mg/mL and sterile filtered. Mice were randomly assigned to receive a single i.p. injection of 10 mg/kg body weight ISO in volumes of 30 to 70 μL. The mice were killed, and muscle tissues were harvested 24 h after ISO injection. A second cohort of mice received up to four injections of 10 mg/kg body weight ISO over the course of 4 wk, delivered once every 7 d. Mice that displayed marked and progressive lethargy indicative of a decline toward death were killed in compliance with the animal use protocol. Four weeks after the final injection, surviving mice were killed, and hearts were harvested. A third cohort of mice received a daily, low-dose (2.5 mg/kg) ISO challenge. ISO was prepared as described above, but at a concentration of 1.5 mg/mL. Mice received a daily i.p. injection of 2.5 mg/kg body weight ISO over the course of 10 d. Dying mice were killed in compliance with the animal use protocol. On the 11th day, mice that survived the challenge were killed and their hearts were collected.

### AAV Vector Production and In Vivo Administration.

The sequence encoding mouse *Pomt1* was synthesized (Genscript, Piscataway, NJ) and then cloned into the AAV backbone under the transcriptional control of the striated muscle-specific creatine kinase (*MCK*) promoter (a gift from Jeffrey Chamberlain at the University of Washington, Seattle). The vector AAV2/9-MCK-Pomt1 was generated by the University of Iowa Viral Vector Core Facility. Pomt1 cKO mice (4 to 8 wk old) received a single, 50 μL injection of the vector solution (3.28 × 10^13^ vg/mL) via the retro-orbital sinus intravenous route.

### Statistics.

All data in the present study are shown as the means ± SD unless noted otherwise. The number of sampled units, *n*, is a single mouse for an experiment (i.e., one mouse is *n* = 1). GraphPad Prism 9 software was used for all statistical analyses. The two-tailed *t* test was used when a single dataset was compared to a separate dataset. The multiple unpaired *t* test with the Holm–Sidak post hoc test was used for age-matched comparisons. The paired *t* test with the Holm–Sidak post hoc test was used when the same animal was examined under two different conditions. Differences were considered significant at a *P*-value less than 0.05.

## Supplementary Material

Appendix 01 (PDF)

## Data Availability

All materials and reagents used in this study are reported in Materials and Methods. All study data are included in the article and/or *SI Appendix*. Requests for further information, resources, or reagents should be directed to and will be fulfilled by the corresponding author: Kevin P. Campbell (kevin-campbell@uiowa.edu).
