# Supplementary material for: Matriglycan maintains t-tubule structural integrity in cardiac muscle
Source: Proc Natl Acad Sci U S A. 2024 May 21;121(22):e2402890121. doi: 10.1073/pnas.2402890121 (PMC11145246; doi:10.1073/pnas.2402890121)
Supplement: Supplementary file 1 — Appendix 01 (PDF) [file pnas.2402890121.sapp.pdf]

PNAS

Supporting Information for

Matriglycan maintains t-tubule structural integrity in cardiac muscle

Jeffrey M. Hord[1], Mary E. Anderson[1], Sally J. Prouty[1], Shelly Melton[1], Zeita Gastel[1], Kathy Zimmerman[3], Robert M. Weiss[2, 3, 4], Kevin P. Campbell[1*]

Kevin P. Campbell
Email: kevin-campbell@uiowa.edu

This PDF file includes:

Figures S1 to S14

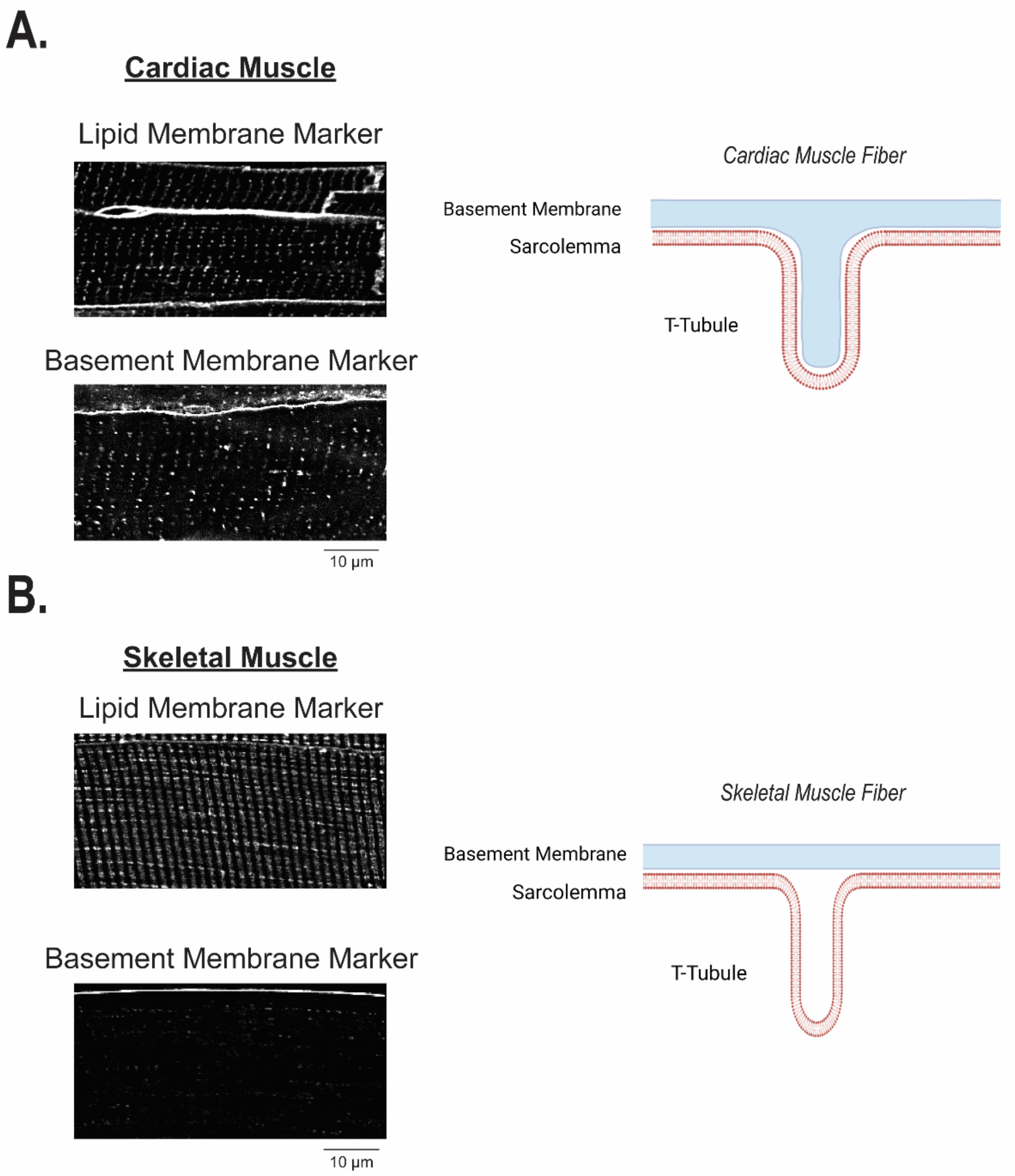

Fig. S1. The presence of basement membrane within the t-tubule lumen is specific to cardiac muscle fibers. A-B Fluorescent labeling of myofiber lipid membranes with FM464-FX and basement membranes with WGA-AlexaFluor488 on cardiac ventricular myofibers **A**, and extensor digitorum longs (EDL) skeletal muscle fibers **B**, from control mice. 120x magnification; Scale bar = 20 μm. Illustrations depict the differences between t-tubules in cardiac muscle and skeletal muscle. The lumen of cardiac muscle t-tubules contains extracellular basement membrane and generally has a greater width compared to that of skeletal muscle t-tubules.

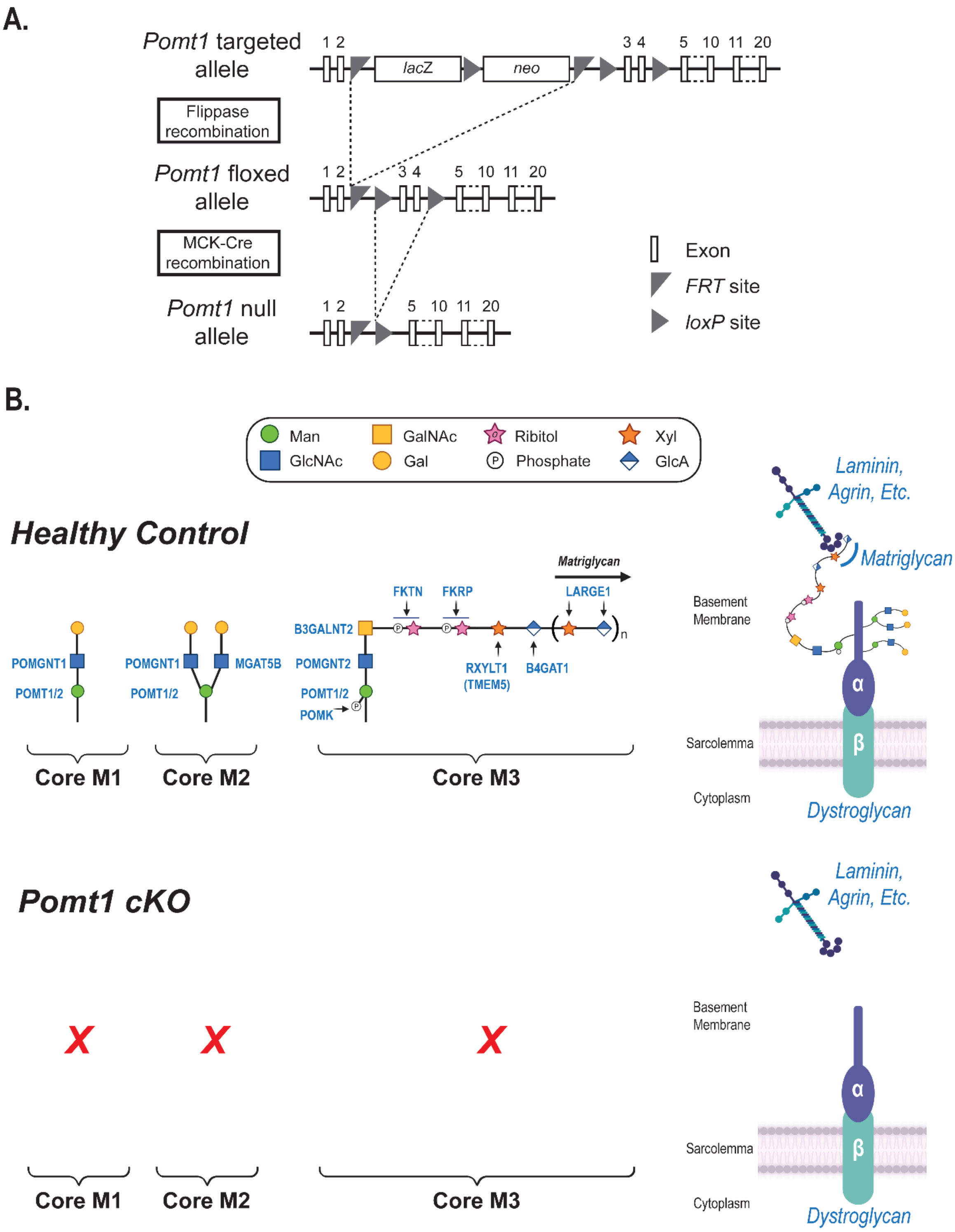

Fig. S2. Generation of the MCK-Cre / floxed Pomt1 mouse line. A, Design of the floxed *Pomt1* mouse line crossed with Cre under the MCK promoter. **B**, Comparison of core M1, M2, and M3 biosynthesis in healthy control mice and Pomt1 cKO mice. Panels on the right illustrate sarcolemma-localized dystroglycan and its interaction with ECM ligands. Abbreviations: Man, mannose; GlcNAc, *N*-acetyl-glucosamine; Gal, galactose; GalNAc, *N*-acetyl-galactosamine; Xyl, xylose; GlcA, glucuronic acid; POMT1/2, protein *O*-mannosyltransferases 1 and 2; POMGNT1, protein *O*-linked mannose *N*-acetyl-glucosaminyltransferase 1; POMGNT2, protein *O*-linked mannose *N*-acetyl-glucosaminyltransferase 2; MGAT5B, mannosyl α1,6-glycoprotein β1,6,-*N*-

acetyl-glucosaminyltransferase; POMGNT2, protein *O*-linked mannose *N*-acetyl-glucosaminyltransferase 2; B3GALNT2, β1,3-*N*-acetylgalactosaminyltransferase 2; POMK, protein *O*-mannose kinase; FKTN, Fukutin; FKRP, Fukutin related protein; RXYLT1, ribitol xylosyltransferase 1; TMEM5, transmembrane protein 5; B4GAT1, β1,4-glucuronyltransferase 1; LARGE1, like-acetyl-glucosaminyltransferase 1.

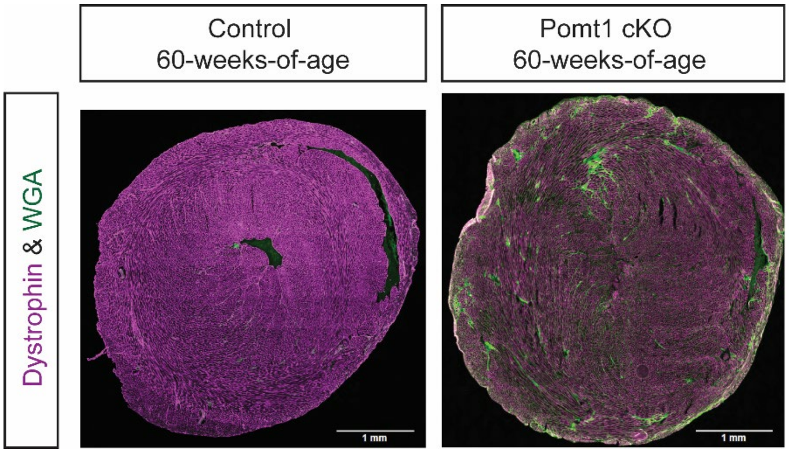

Fig. S3. Immunofluorescence on left ventricular cross-sections of 60-week-old control and Pomt1 cKO mice. Scale bar = 1 mm.

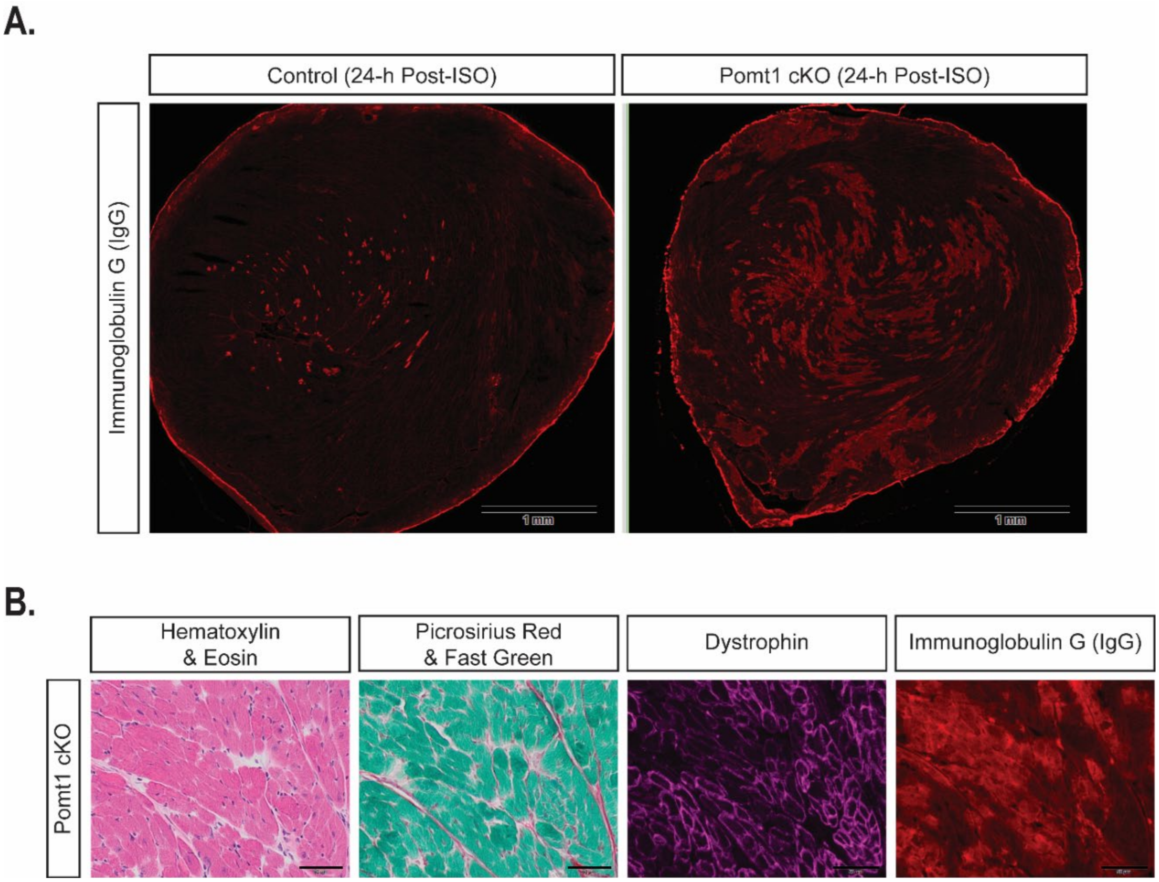

Fig. S4. Isoproterenol-induced stress causes injury to cardiomyocytes from Pomt1 cKO mice. Sixteen- to 36-week-old control and Pomt1 cKO mice received intraperitoneal (i.p.) injection of isoproterenol (ISO; 10 mg/kg body weight). **A**, Immunofluorescence of hearts from control or Pomt1 cKO mice 24-h after injection. Scale bar = 1 mm. **B**, Histological analysis of muscle from the cardiac ventricle isolated from Pomt1 cKO mice 24-h post-ISO injection. Cryosections are stained with Hematoxylin and Eosin, Picrosirius red and Fast Green, anti-dystrophin, and Immunoglobulin G (IgG). Scale bar = 50 μm.

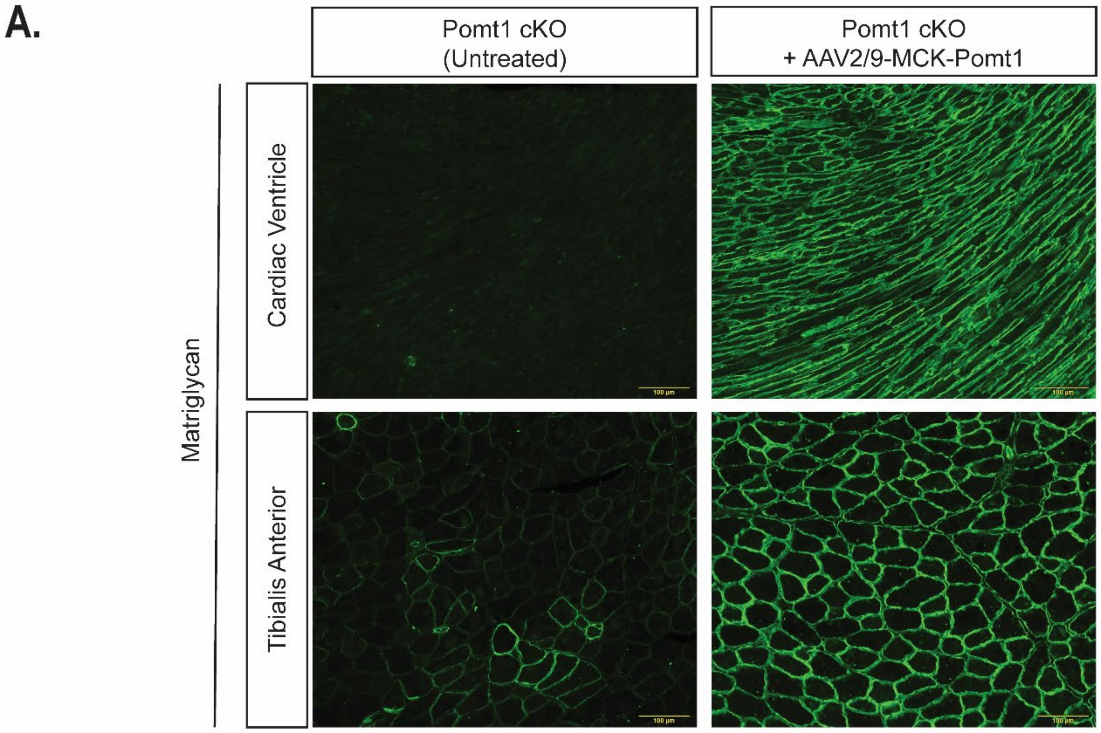
A.
Pomt1 cKO
(Untreated)
Pomt1 cKO
+ AAV2/9-MCK-Pomt1
Matriglycan
Cardiac Ventricle
Tibialis Anterior

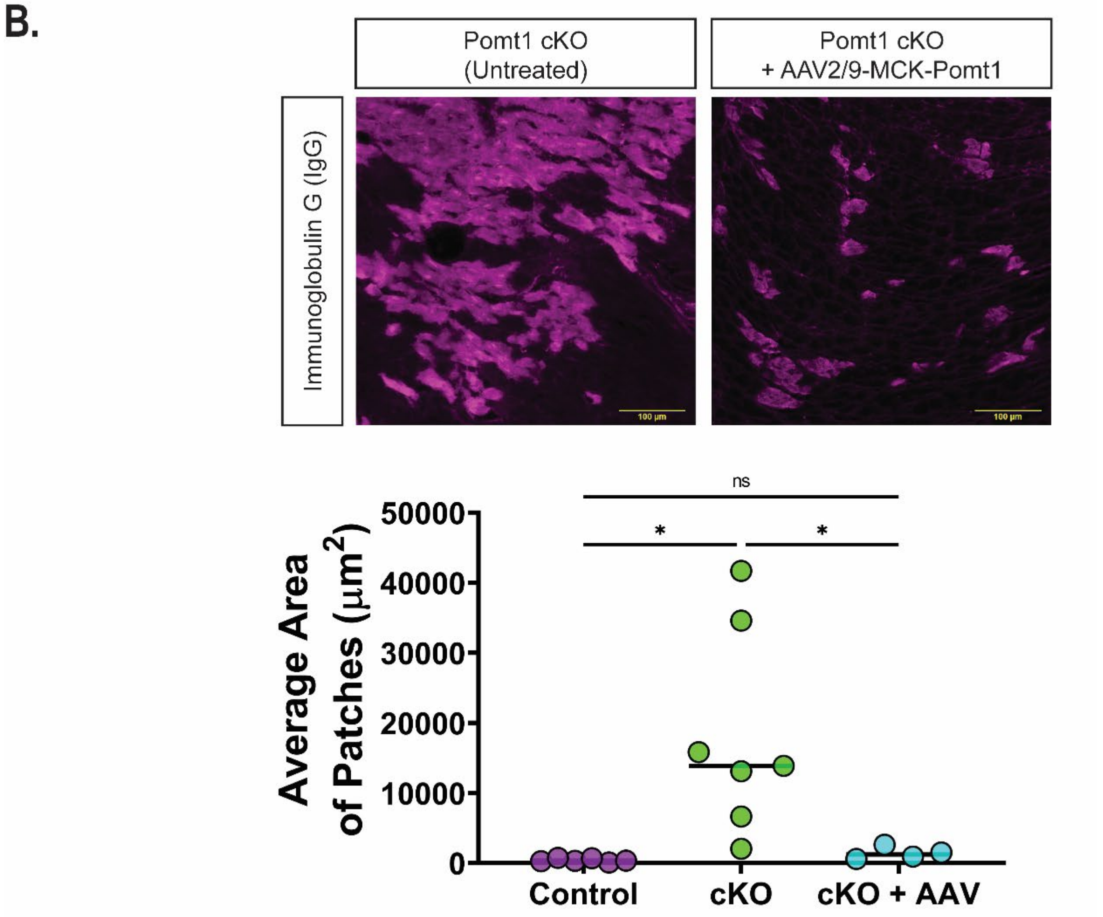
B.
Pomt1 cKO
(Untreated)
Pomt1 cKO
+ AAV2/9-MCK-Pomt1
Immunoglobulin G (IgG)
100 µm
100 µm
ns
*
*
Average Area of Patches (µm2)
50000
40000
30000
20000
10000
0
Control
cKO
cKO + AAV

Fig. S5. Administration of AAV2/9-MCK-Pomt1 to Pomt1 cKO mice restores matriglycan levels and improves cardiomyofiber protection against contraction-induced damage. Pomt1 cKO mice were untreated or were injected with AAV2/9-MCK-Pomt1. **A**, Immunofluorescence of cardiac ventricles and tibialis anterior muscles to detect matriglycan (IIH6 antibody). Scale bar = 100 μm. Control, Pomt1 cKO, and Pomt1 cKO + AAV2/9-MCK-Pomt1 mice received an injection of ISO (10 mg/kg body weight i.p.) and their hearts were harvested 24-h later. **B**, *Top*, immunofluorescence of intra-myofiber IgG. *Bottom*, quantification of the average size area of IgG-labeled myofiber patches in control, Pomt1 cKO mice, and Pomt1 cKO mice that received AAV2/9-MCK-Pomt1. Histopathology was performed in male and female mouse hearts (Control, n = 6; Pomt1 cKO, n = 7; Pomt1 cKO + AAV2/9-MCK-Pomt1, n = 4). Statistical analyses were performed by unpaired t-test with Holm-Sidak post-hoc. Data expressed as mean ± standard deviation. * > 0.05.

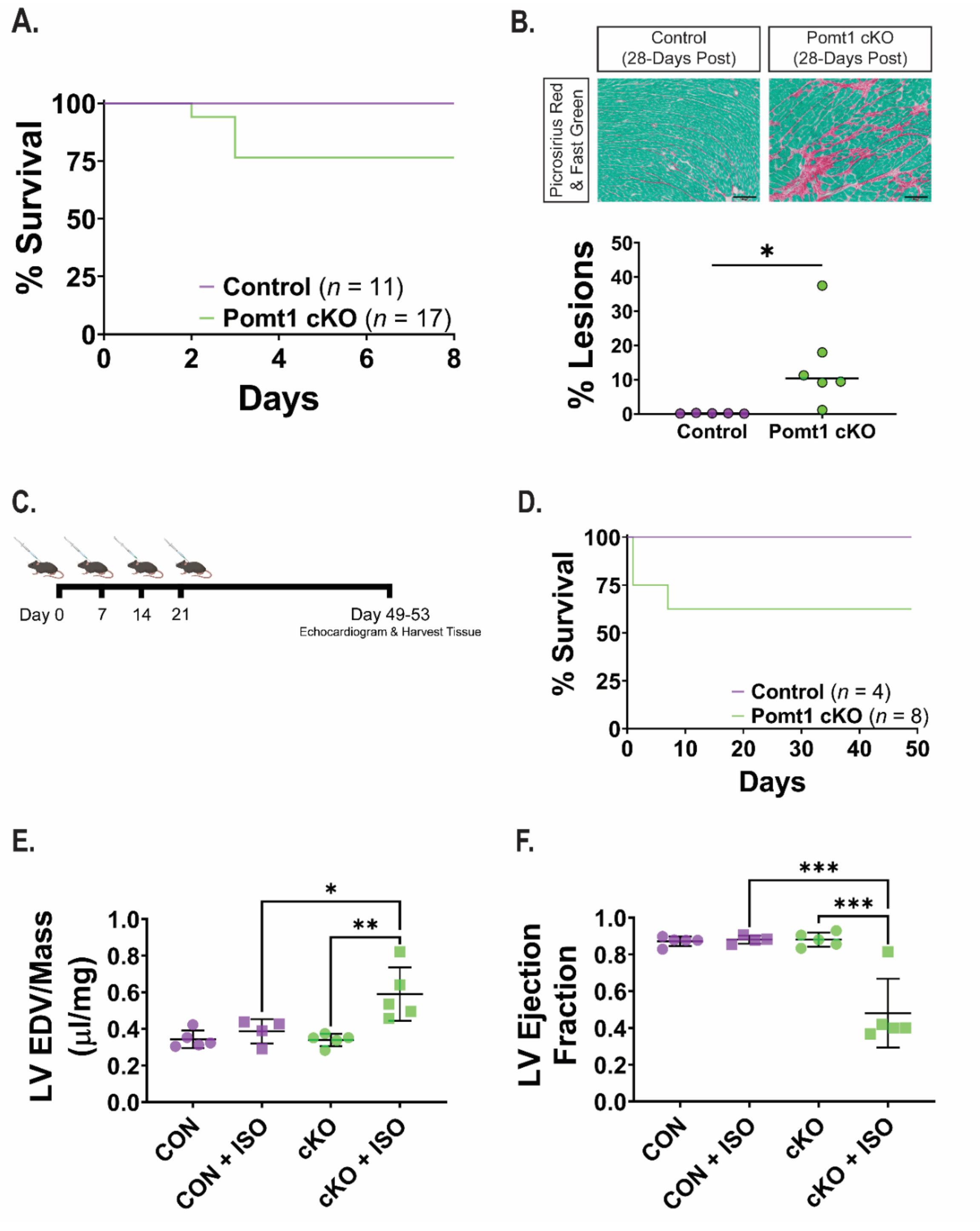

Fig. S6. Catecholamine-induced stress promotes progressive cardiomyopathy in Pomt1 cKO hearts. A-B Control and Pomt1 cKO mice were injected with a single bolus of ISO (10 mg/kg body weight). **A**, Kaplan-Meier survival curve of controls and Pomt1 cKO mice. Experiments were performed with mice of both sexes. Controls, *n* = 11; Pomt1 cKO, *n* = 17. **B**, Histological analysis of cardiac tissues 28 days after injection. Cardiac sections were stained with Picrosirius red and Fast Green. Scale = 100 µm. Quantification of ventricular fibrosis of tissue stained with Picrosirius red. Experiments were performed with mice of both sexes. Controls, *n* = 5; Pomt1 cKO, *n* = 6. Statistical analyses were performed with unpaired t-test with Holm-Sidak post-hoc. Data expressed

as mean ± standard deviation. * = 0.03 **C**, Schematic of experimental design to provide repeated ISO injections once every seven days over a 28-day period in control and Pomt1 cKO mice. **D**, Kaplan-Meier survival curve of controls and Pomt1 cKO mice injected as in **C**. **E-F** Echocardiographic analysis to determine **E**, left ventricle (LV) end diastolic volume per mass (LV EDV/Mass) and **F**, LV ejection fraction in mice treated as in **C**. Controls, *n* = 4; Controls + ISO, *n* = 4; Pomt1 cKO, *n* = 5; Pomt1 cKO + ISO, *n* = 5 (began with *n* = 8). Statistical analyses were performed with one-way ANOVA with Tukey's post-hoc. Data expressed as mean ± standard deviation. * = 0.02; ** = 0.002; *** <0.0001.

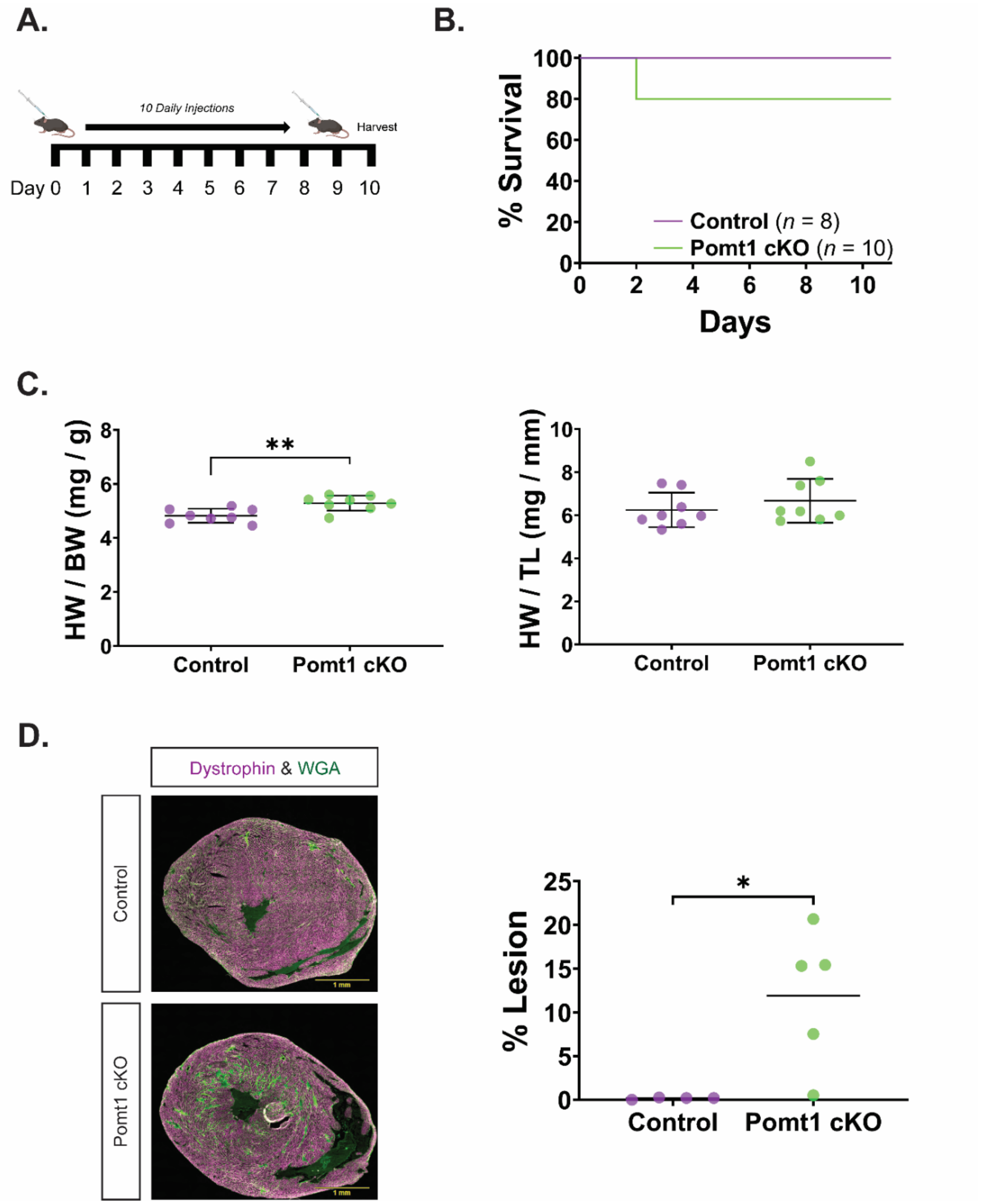

Fig. S7. Daily, low dose β-adrenergic challenge leads to death and disrupts the ventricular morphology in Pomt1 cKO mice. A, Schematic of experimental design. Control and Pomt1 cKO mice received repeated i.p. injections of ISO (2.5 mg/kg body weight). **B**, Kaplan-Meier survival curve of control and Pomt1 cKO mice treated as in **A**. (Controls, n = 8; Pomt1 cKO, n = 10). **C**, Heart weight normalized to body weight (*left panel*) and heart weight normalized to tibial length (*right* panel) of mice in **B**. **, p = 0.0037, as determined by unpaired two-tailed t-test. **D**, Immunofluorescence on ventricular cross-sections from mice in **B**. WGA-488 (*green*) was used as a marker of the extracellular matrix and dystrophin (*purple*) was used as a myocyte membrane marker. Scale bar = 1 mm. Graph displays quantification of ventricular lesions. Experiments were

performed with mice of both sexes. Controls, *n* = 4; Pomt1 cKO, *n* = 5. Statistical analyses were performed with unpaired t-test with Holm-Sidak post-hoc. Data expressed as mean ± standard deviation. *, $p = 0.0222$.

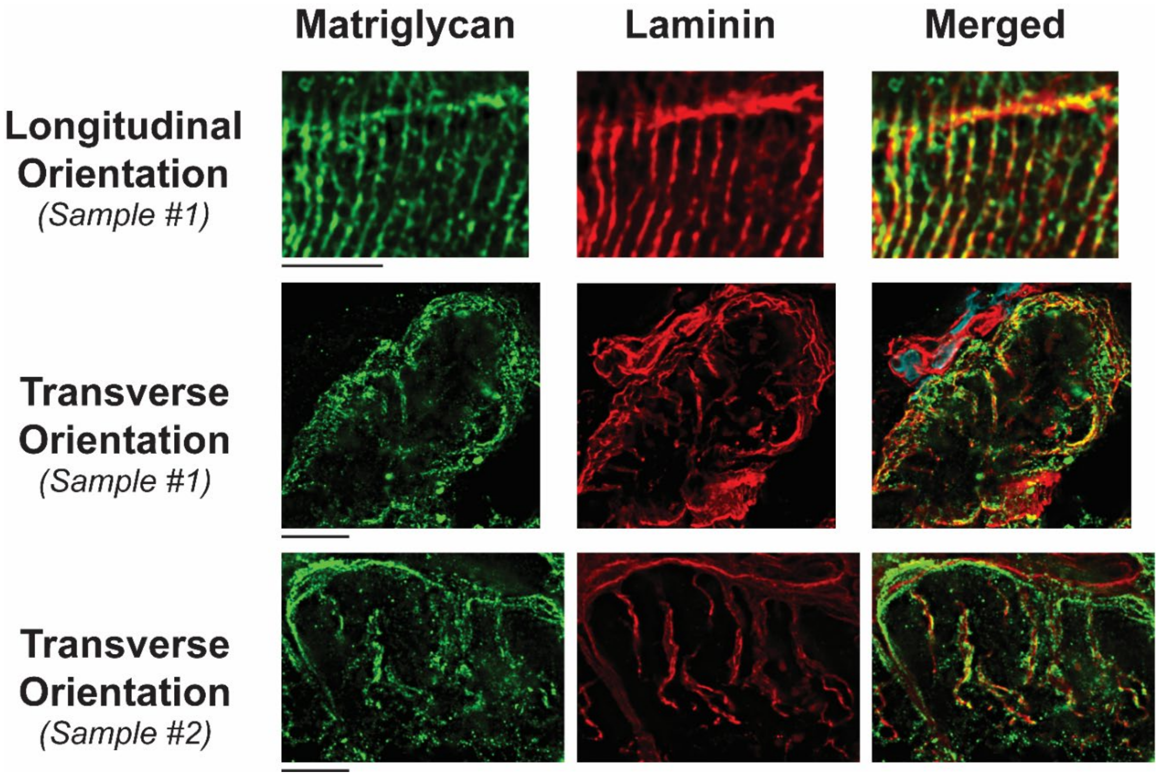

Fig. S8. Presence of matriglycan in human cardiac muscle t-tubule membranes. Immunofluorescence on frozen human heart cryosections to detect the presence of matriglycan and extracellular matrix laminin in cardiac muscle fibers. *Top row* shows matriglycan and laminin immunofluorescence in longitudinally oriented cardiac muscle fibers. Scale bar = 5 µm. *Middle* and *bottom rows* show matriglycan and laminin immunofluorescence in transverse oriented cardiac muscle fibers. Scale bars = 10 µm. Results were collected from two human heart samples.

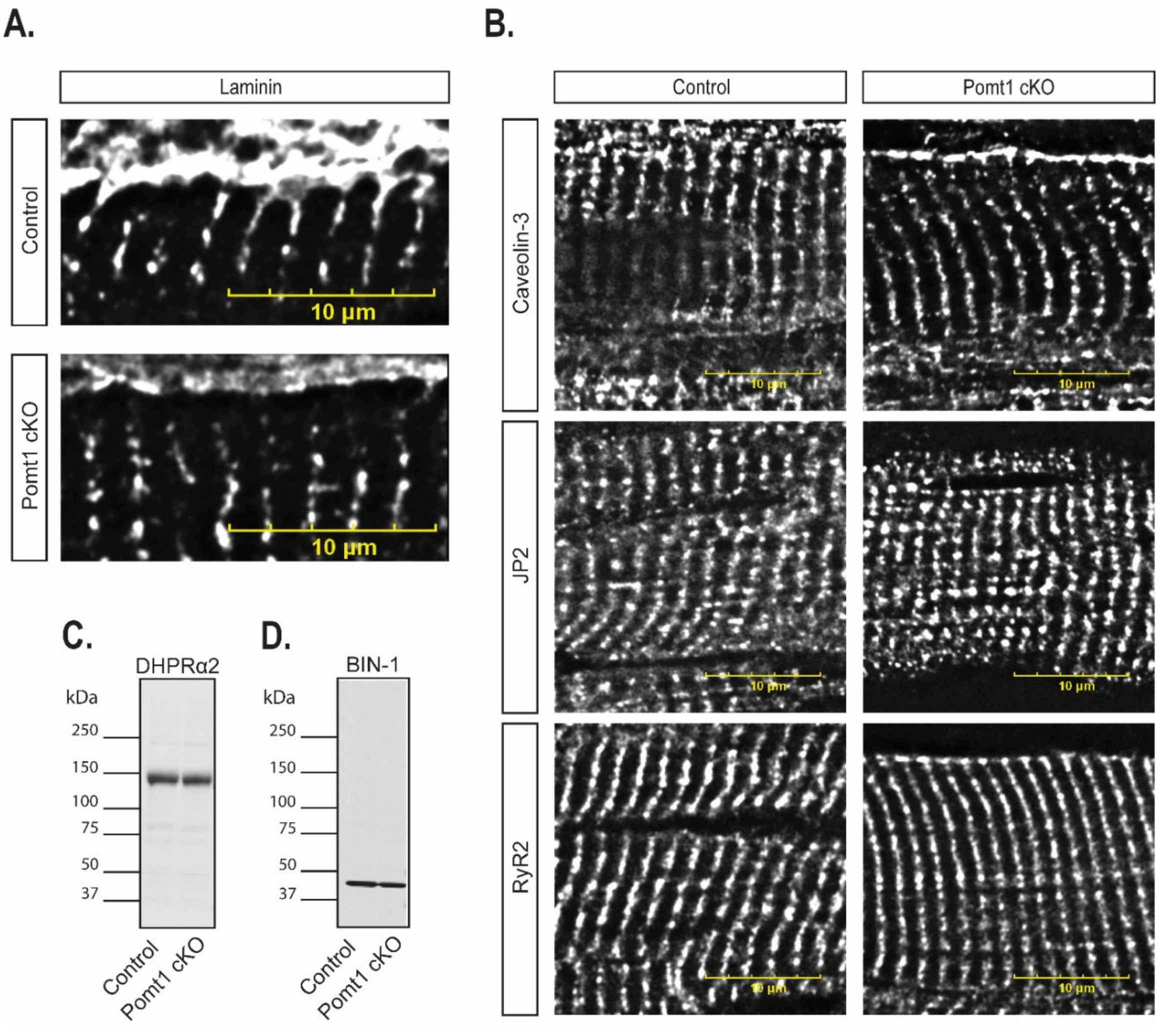

Fig. S9. Development of the t-tubule network does not require *O*-mannosylated α-DG. A-B Immunofluorescence of cryosections of ventricles from control and Pomt1 cKO mice to detect basement membrane protein laminin **A**, membrane localized protein caveolin-3 **B**, and junctional dyad proteins junctophilin 2 (JP2) and ryanodine receptor 2 (RyR2). Scale bar = 10 µm. **C-D** Immunoblot analysis of WGA-enriched control and Pomt1 cKO cardiac muscle t-tubule membrane proteins dihydropyridine receptor α-2 (DHPRα2; **C**) and BIN-1 **D**.

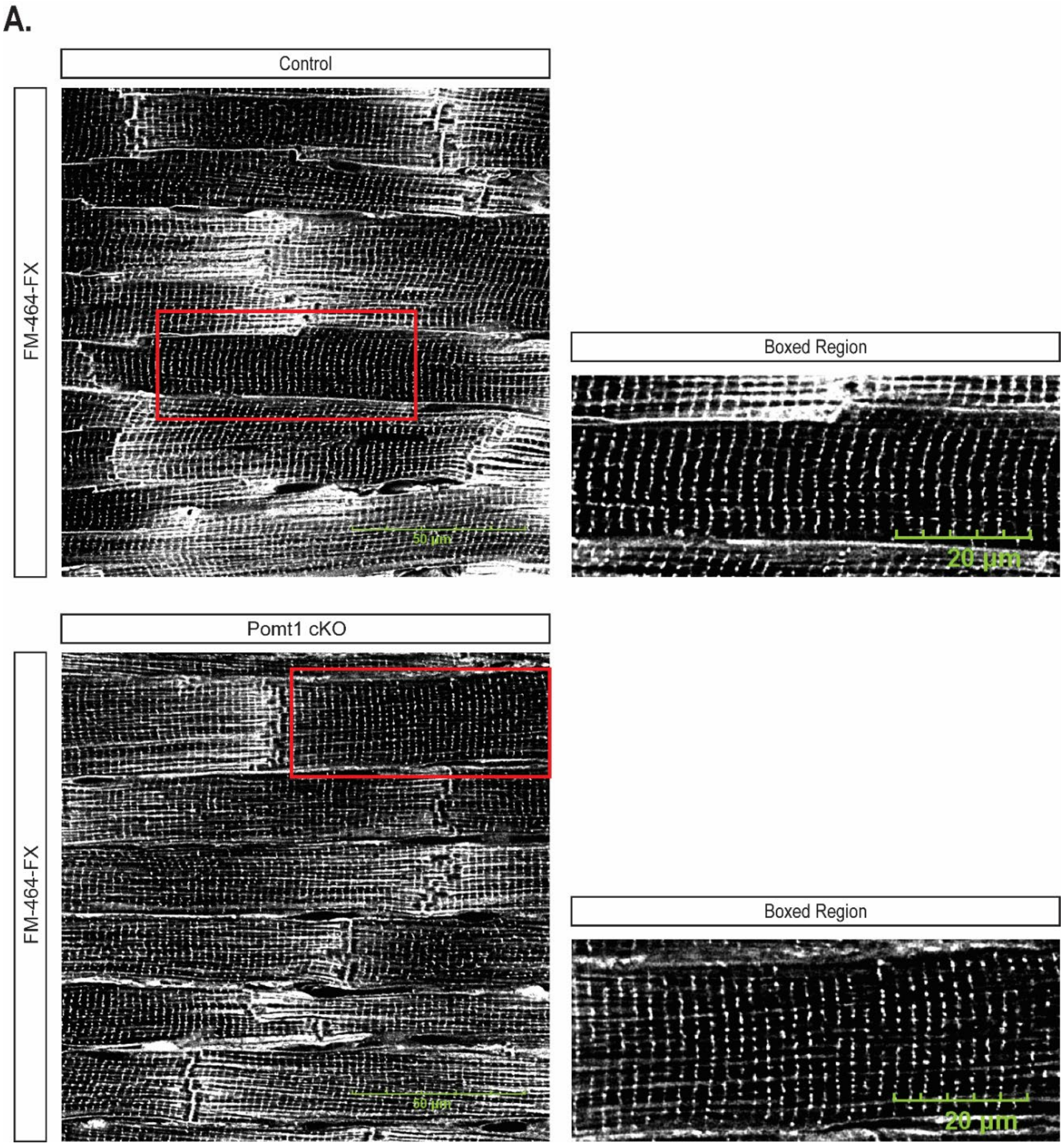

Fig. S10. FM 464-FX images shown on the left were captured at 90x magnification. Scale bar = 50 µm. Boxed regions (red rectangular box) highlight the region of the myocardium that is shown in the images on the right (as shown in **Fig. 4C**). Scale bar = 20 µm.

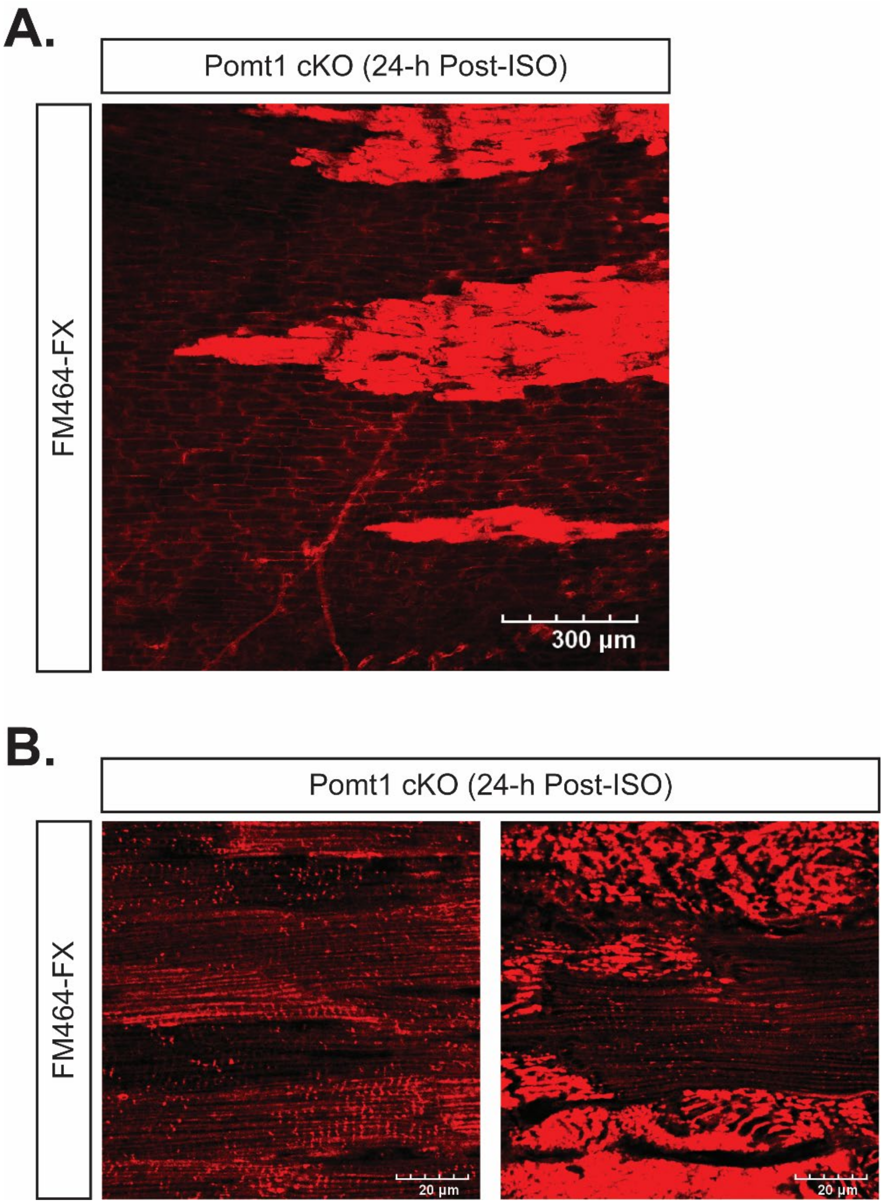

Fig. S11. Lipophilic dye, FM464-FX, infiltrates myocytes that suffered catastrophic sarcolemma damage. A, Low magnification (10x) examination identified regions of fibers that displayed myocyte uptake of FM464-FX, while many of the cardiac fibers were spared of catastrophic membrane damage in stressed Pomt1 cKO hearts. Scale bar = 300 µm. **B**, Variations in myocyte damage are shown. *Left panel* displays cardiac fibers that did not accumulate intra-myocyte FM464-FX but were susceptible to t-tubule disruption. *Right panel* shows cardiac fibers on the border of an ischemic region. Myocytes that were infiltrated with FM464-FX dye are intensely red, whereas fibers that avoided catastrophic membrane damage did not show myocyte infiltration of the dye.

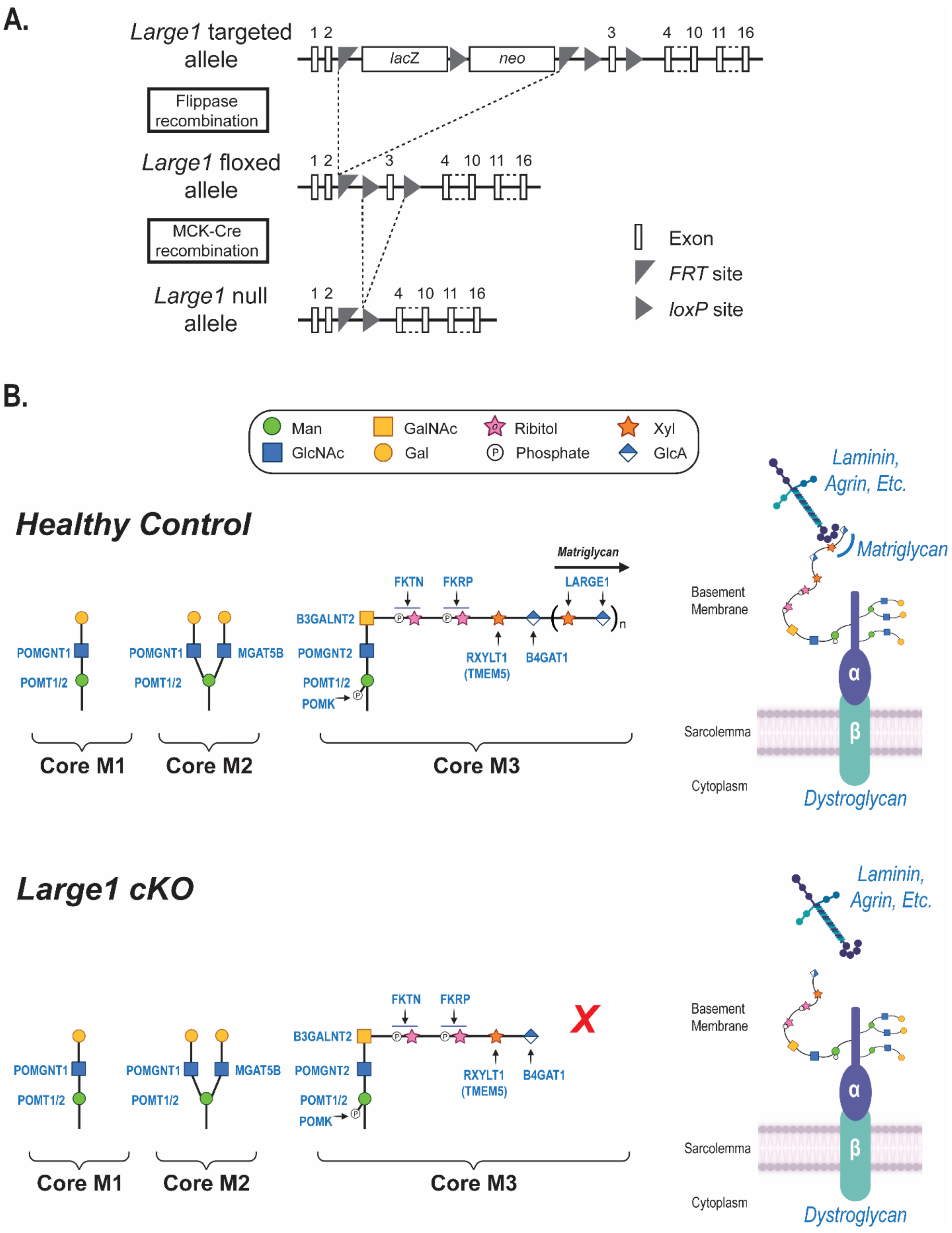

Fig. S12. Generation of the MCK-Cre / floxed Large1 mouse line. A, Design of the floxed *Large1* mouse line crossed with Cre under the MCK promoter. **B**, Comparison of core M1, M2, and M3 biosynthesis in healthy control mice and Large1 cKO mice. Panels on the right illustrate sarcolemma-localized dystroglycan and its interaction with ECM ligands. Abbreviations: man, mannose; GlcNAc, *N*-acetyl-glucosamine; Gal, galactose; GalNAc, *N*-acetyl-galactosamine; Xyl, xylose; GlcA, glucuronic acid; POMT1/2, protein *O*-mannosyltransferases 1 and 2; POMGNT1,

protein *O*-linked mannose *N*-acetyl-glucosaminyltransferase 1; POMGNT2, protein *O*-linked mannose *N*-acetyl-glucosaminyltransferase 2; MGAT5B, mannosyl α1,6-glycoprotein β1,6,-*N*-acetyl-glucosaminyltransferase; POMGNT2, protein *O*-linked mannose *N*-acetyl-glucosaminyltransferase 2; B3GALNT2, β1,3-*N*-acetylgalactosaminyltransferase 2; POMK, protein *O*-mannose kinase; FKTN, Fukutin; FKRP, Fukutin related protein; RXYLT1, ribitol xylosyltransferase 1; TMEM5, transmembrane protein 5; B4GAT1, β1,4-glucuronyltransferase 1; LARGE1, like-acetyl-glucosaminyltransferase 1.

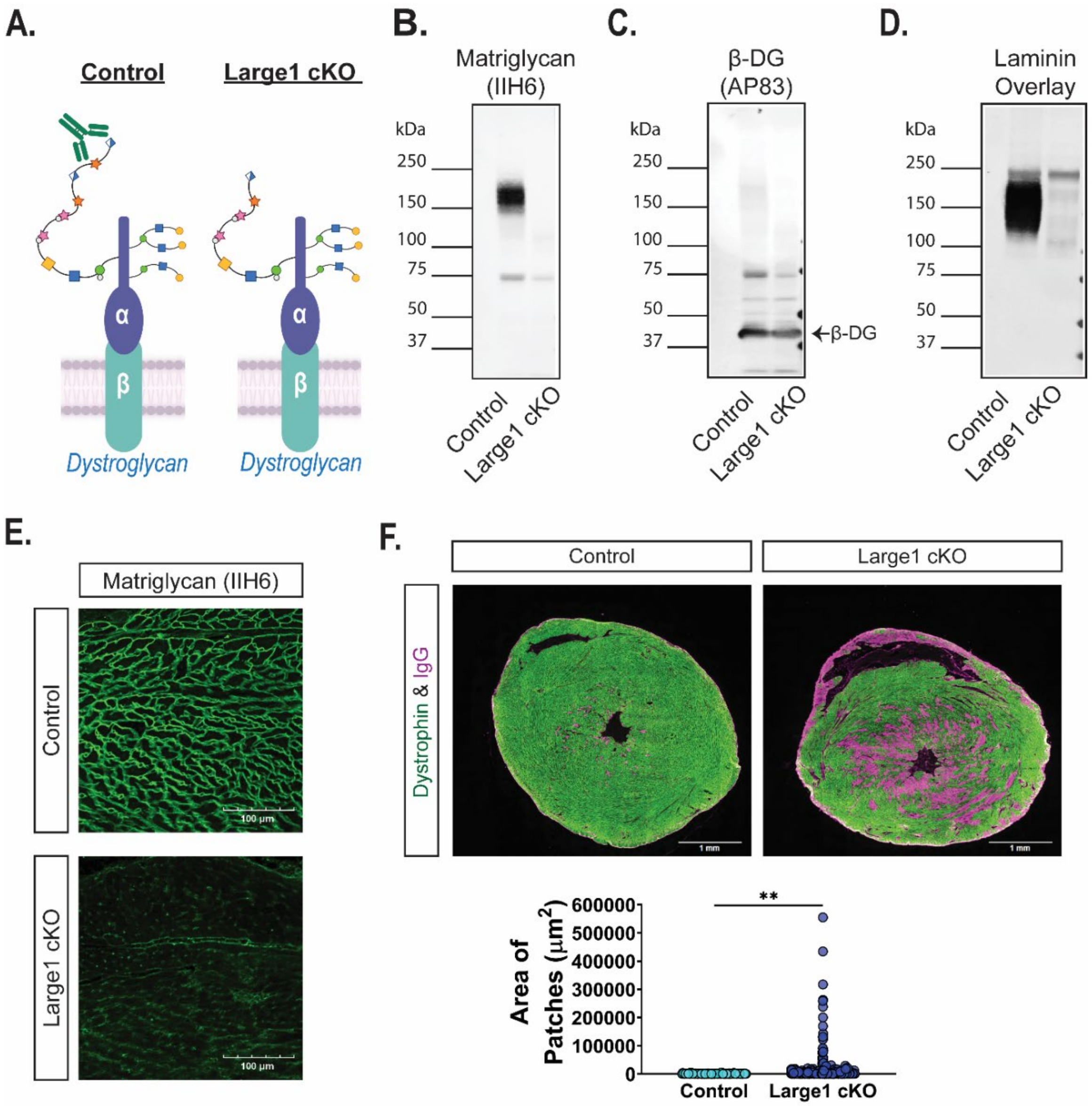

Fig. S13. Cardiac muscle matriglycan is required to prevent stress-induced membrane damage. **A**, DG core M glycans in control and Large1 cKO. **B-D** immunoblots of ventricle muscle to detect **B**, matriglycan; **C**, β-DG; and **D**, laminin binding. Experiments were performed on two samples per group. **E**, Immunofluorescence of ventricles to detect matriglycan (n = 3 controls and n = 3 Large1 cKO mice). Scale bar = 100 µm. Isoproterenol (ISO; 10 mg/kg body weight) was administered to promote an acute bout of increased cardiac workload. Mice were sacrificed 24-h post-injection. **F**, Ventricular cross-sections were used to assess cardiomyofiber damage as detected by intramyocyte IgG. Scale bar = 1 mm. Quantification of the area of individual patches of damaged fibers. Unpaired t-test with Holm-Sidak post-hoc were performed. Data expressed as mean ± standard deviation. ** p = 0.007.

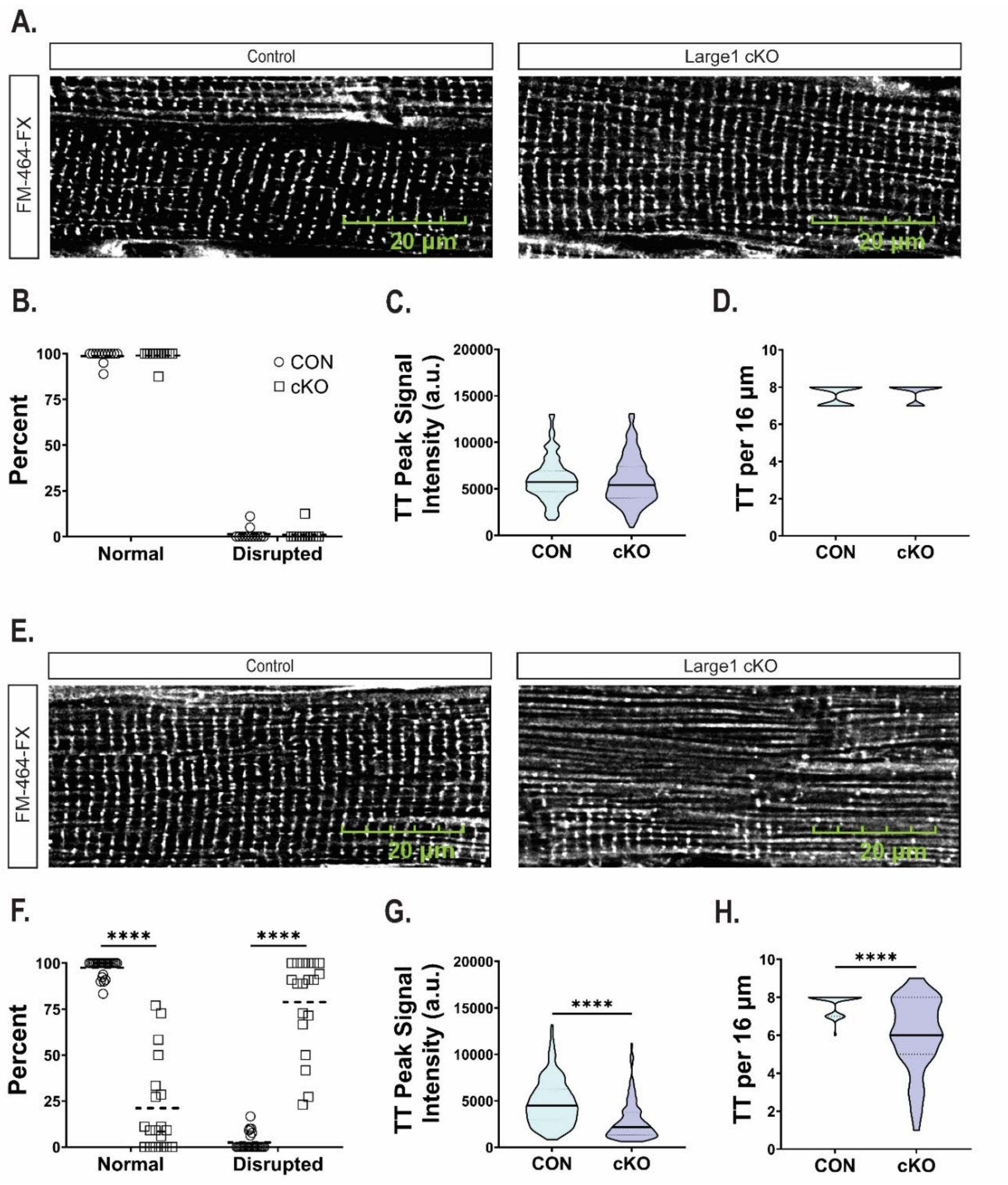

Fig. S14. Cardiac muscle matriglycan is essential to prevent stress-induced t-tubule damage. A, Labeling of control and Large1 cKO whole left ventricles stained with FM 464-FX under baseline conditions. Scale bar = 20 µm. **B**, Percentage of myofibers that showed either normal or disrupted patterns of t-tubules. **C**, Line scan analysis to determine the peak signal intensity of FM 464-FX labeled t-tubules (TT). **D**, Number of t-tubules observed within a 16 µm regions. Image analysis was performed on hearts of mice from both sexes. Unpaired t-test with Holm-Sidak post-hoc were performed. Data expressed as mean ± standard deviation. **E**, Labeling of control and Large1 cKO whole left ventricle stained with FM 464-FX 24-h after ISO challenge. **F-H**, Quantification as performed in **B-C**. Image analysis was performed on hearts from both sexes. Unpaired t-test with Holm-Sidak post-hoc were performed. Data expressed as mean ± standard deviation. **** p = 0.0001.
